# In-depth transcriptome characterization uncovers distinct gene family expansions for *Cupressus gigantea* important to this long-lived species’ adaptability to environmental cues

**DOI:** 10.1186/s12864-019-5584-6

**Published:** 2019-03-13

**Authors:** Shan-Shan Zhou, Zhen Xing, Hui Liu, Xian-Ge Hu, Qiong Gao, Jie Xu, Si-Qian Jiao, Kai-Hua Jia, Yu Qing Jin, Wei Zhao, Ilga Porth, Yousry A. El-Kassaby, Jian-Feng Mao

**Affiliations:** 1 Beijing Advanced Innovation Center for Tree Breeding by Molecular Design, National Engineering Laboratory for Tree Breeding, Key Laboratory of Genetics and Breeding in Forest Trees and Ornamental Plants, Ministry of Education, College of Biological Sciences and Technology, Beijing Forestry University, Beijing, 100083 China; 2 Resources & Environmental College, Tibet Agriculture & Animal Husbandry University, Linzhi, 860000 Tibet China; 3 Départment des Sciences du Bois et de la Forêt, Avenue de la Médecine, Université Laval, Québec, QC, G1V 0A6 Canada; 4 Department of Forest and Conservation Sciences, Faculty of Forestry, University of British Columbia, Vancouver, BC V6T 1Z4 Canada

**Keywords:** *Cupressus gigantea*, De novo transcriptome assembly, Ecology, Gene family evolution, Gene functional annotation

## Abstract

**Background:**

*Cupressus gigantea*, a rare and endangered tree species with remarkable medicinal value, is endemic to the Tibetan Plateau. Yet, little is known about the underlying genetics of the unique ecological adaptability of this extremely long-lived conifer with a large genome size. Here, we present its first de novo and multi-tissue transcriptome in-depth characterization.

**Results:**

We performed Illumina paired-end sequencing and RNA libraries assembly derived from terminal buds, male and female strobili, biennial leaves, and cambium tissues taken from adult *C. gigantea*. In total, large-scale high-quality reads were assembled into 101,092 unigenes, with an average sequence length of 1029 bp, and 6848 unigenes (6.77%) were mapped against the KEGG databases to identify 292 pathways. A core set of 41,373 genes belonging to 2412 orthologous gene families shared between *C. gigantea* and nine other plants was revealed. In addition, we identified 2515 small to larger-size gene families containing in total 9223 genes specific to *C. gigantea*, and enriched for gene ontologies relating to biotic interactions. We identified an important terpene synthases gene family expansion with its 121 putative members.

**Conclusions:**

This study presents the first comprehensive transcriptome characterization of *C. gigantea*. Our results will facilitate functional genomic studies to support genetic improvement and conservation programs for this endangered conifer.

**Electronic supplementary material:**

The online version of this article (10.1186/s12864-019-5584-6) contains supplementary material, which is available to authorized users.

## Background

*Cupressus gigantea* W.C. Chen et L.K. Fu, also called Tibetan cypress or giant cypress, is a rare and vulnerable conifer tree species endemic to the Tibetan Plateau. Natural populations are scarce for this species, which led *C. gigantea* to be listed on the Chinese National Protection List of Wild Plant (Class I) [1]. As a rare conifer, *C. gigantea* grows sparsely in the narrow dry valleys of the Yarlung Zangbo and Nyang Rivers on the Tibetan Plateau at an elevation band between 3000 and 3400 m [2]. *C. gigantea* is an excellent timber species with high wood density, straight grain, and radial uniformity; an average tree height of about 20-30 m, with few trees reaching up to 50 m; the diameter can reach up to 6 m and the age of the trees can reach more than 2600 years, making *C. gigantea* one of the long-lived endemic cypresses in China [2]. According to the International Union for Conservation of Nature (IUCN) Red List categories of threatened species, *C. gigantea* has been classified as a vulnerable species due to highly disturbed distribution leading to serious populations reduction [3]. Therefore, based on this special status, *C. gigantea* not only represents important timber and ornamental values, but also great scientific value in terms of ecology and conservation biology, regarding the species’ adaptation to an extreme environment due to the unique geography.

*Cupressus gigantea* has attracted wide interest and has been increasingly studied since it was described as a species by 1975 [4]. Previous studies focused on its geographic distribution [5], photosynthetic capacity [6], and its unique ecology [7] and community characteristics [8] and phylogenetic status [9]. However, these studies did not address the species’ molecular genetics probably due to *C. gigantea*’s high genetic load [10]. Only recently, Li and co-workers isolated and characterized 16 polymorphic microsatellites from *C. gigantea* using paired-end Illumina shotgun sequencing [11]. Subsequently, the species’ complete chloroplast genome was determined [12]. However, these studies did not provide any functional genetic determination of the extreme adaptive potential present within *C. gigantea*. The mining of genes related to adaptive mechanisms such as those involving stress resistance superfamily genes is indispensable to decipher the genetic underpinnings of adaptive phenotypic traits. Using this information in population-wide genetic screens has the potential to accelerate formulations for effective conservation strategies concerning this vulnerable conifer species whose distribution is restricted to the Qinghai-Tibetan Plateau.

Plants have evolved well-orchestrated resistance mechanisms to defend themselves against various environmental pressures [13]. The expression of stress resistance superfamily genes is stimulated in order to render various protective effects to the plant under adverse environmental circumstances. Universal stress proteins (USPs) constitute a natural biological defense mechanism by providing general “stress endurance.” The USP domain contains a protein structure originally identified from *Escherichia coli* as USPA (universal stress protein A) because of its prominence in the stationary phase of bacteria growth. These genes function as regulators of cell survival under heat, starvation, and other biotic or abiotic stresses [14–16]. An additional group of stress molecules involve leucine-rich repeat receptor-like protein kinases (LRR-RLKs) that represent the largest group of RLKs; they mainly contain three functional domains: an extracellular domain (ECD), an intracellular kinase domain (KD), and a transmembrane (TM) domain. Previous studies indicated that LRR-RLK genes play crucial roles in meristematic growth, secondary growth, response to environmental stimuli, bacterial pathogens, and necrotrophic fungi and viruses [17–22]. Furthermore, terpenoids, usually constitute a very large and structurally diverse group of natural products and play main roles in plant defenses and stress resistance [23]. The majority of the terpenoids found in plants so far, have proven functions in plant defenses [24]. Overall, *Eucalyptus grandis* seems to have the largest number (113) of putatively functional terpene synthase (TPS) genes compared to other sequenced plant genomes [25]. Hence, research regarding such defense related gene families will enable better understanding of the diversity of defense genes of a plant species and, at the same time, gene-family phylogenetic analysis will also help to infer gene functional characterizations.

In the present study, we performed de novo transcriptome assembly from terminal buds, microstrobili, female strobili, biennial foliage, and cambial tissues originating from adult *C. gigantea*. We assembled this transcriptome to annotate transcripts using available information in public databases, further categorized for biological functions and pathways, and characterized the diversity and evolutionary history of genes involved in plant stress responses. This represents the first comprehensive description of the global *C. gigantea* transcriptome to date. These new resources will contribute substantially to future functional genomic studies and conservation programs for this endangered species.

## Results

### RNA-seq and de novo transcriptome assembly

In total, the paired-end sequencing yielded 153,140,282 raw read pairs. We initially evaluated the raw read base quality (Additional file 1: Figure S1), trimmed poor-quality bases, and removed all poor-quality reads with Trimmomatic (version 0.36) software [26] with default parameter settings (Additional file 2: Figure S2). After having removed the adaptors and all low quality sequences, the total number of the clean reads amounted to 144,175,052 reads (94% of all initial reads). Next, de novo assembly using Trinity [27] produced a total of 135,542 contigs (103,584,408 bp in total, with mean length being 764 bp and with 18,728 bp the longest read length (Additional file 3: Table S1). These contigs (ordered sequences) were then joined into scaffolds. Subsequently, 102,553 scaffolds (104,432,740 bp in total length) were obtained, with a mean length of 1018 bp and the longest length being 23,390 bp. Subsequently, we extracted unigenes from the assembly obtained with Trinity. A total of 101,092 unigenes (104,109,640 bp) were obtained, the average length was 1029 bp with the longest being 25,331 bp. The unigenes length’s distributions are shown in Additional file 4: Figure S3, and the N50 score for unigenes was 1508 bp.

### Annotation and further functional classification of the gene space in *C. gigantea*

All 101,092 assembled unigenes were searched against Nr, KOG, GO, KEGG and Swiss-Prot protein databases using BLASTx with a 1E-5 E-value cutoff (Additional file 5: Table S2 and Additional file 6: Table S3). Functional annotation of the unigenes against these protein databases revealed a total of 33,302 (32.94% of the total) unigenes (Additional file 7: Table S4) with corresponding annotations in Nr and 24,078 (23.81%) unigenes (Additional file 8: Table S5) showed significant similarity to known proteins in the Swiss-Prot protein database. The top-scoring BLASTx hits against the Nr protein database revealed strongest similarities to *Picea sitchensis* (24.27%), *Amborella trichopoda* (9.69%), and *Vitis vinifera* (7.36%) genes.

Querying against Swiss-Prot containing proteins, we found 24,078 unigenes with matching hits, accounting for 23.81% of the total annotations (Additional file 8: Table S5). The top-ten most similar species from Swiss-Prot results were *Arabidopsis thaliana* (48.74%), *Oryza sativa subsp. japonica* (5.37%), *Schizosaccharomyces pombe* (strain 972/ATCC 24843) (3.89%), *Nicotiana tabacum* (3.64%), *Saccharomyces cerevisiae* (strain ATCC 204508 / S288c) (3.43%), *Homo sapiens* (3.02%), *Mus musculus* (3.02%), *Drosophila melanogaster* (2.03%), *Dictyostelium discoideum* (1.49%) and *Nicotiana glutinosa* (1.49%).

The functional classification of GO categories was carried out with Blast2GO. A total of 28,087 unigenes (27.78% of all unigenes) matched with classifications of 3 GO functional categories: biological process (BP), cellular component (CC) and molecular function (MF) (Additional file 9: Figure S4). There were 26 subcategories for biological process, including metabolic processes (15,734, 15.56%), followed by cellular processes (15,693, 15.52%) and single-organism processes (13,502, 13.36%). CC was divided into 17 subcategories including cell part (42,520, 42.06%), followed by cell (21,262, 21.03%) and organelle (16,223, 16.05%). 18 subcategories were found under MF, including the term catalytic activity (20,356, 20.14%), followed by binding (7,878, 7.79%) and nucleic acid binding transcription factor activity (5,145, 5.09%).

A total of 16,600 unigenes (16.42%) matched entries in the KOG database [28] providing 18,810 functional annotations (Additional file 10: Figure S5). Among all 25 corresponding KOG categories, the largest category was signal transduction mechanisms (2,714, 2.68%), followed by general function prediction (2,292, 2.27%), posttranslational modification, protein turnover, chaperones (1,769, 1.75%), unknown function (1,123, 1.11%), carbohydrate transport and metabolism (1,040, 1.03%) and other categories with a percentage lower than 1%. The smallest category for KOG annotations was cell motility, containing only 5 unigenes as a result.

All unigenes from the *C. gigantea* transcriptome assembly were subjected to KASS (KEGG Automatic Annotation Server) pathways annotation (Additional file 11: Table S6). We found 6,848 unigenes (6.77%) matching with a total of 292 pathways. The overrepresented pathways were metabolic pathways (2,067 unigenes, 30.18%) and biosynthesis of secondary metabolites (1,483, 21.66%). These pathways provide a valuable resource for investigating specific molecular processes in *C. gigantea*. Furthermore, 375 unigenes involved environmental adaptive pathways, which contained the following five pathways: plant-pathogen interaction (211 unigenes), circadian rhythm plant (24), circadian rhythm-fly (8), circadian entrainment (73), and circadian rhythm (18). These pathways may be related to controlling plant physiology. For example, such physiological activities help to adapt to environmental changes by controlling the circadian rhythm [29]. Here, metabolic pathway of the terpenoid backbone biosynthesis for the unigenes identified in *C. gigantea* is shown in Additional file 12: Figure S6.

### Expansion/contraction of gene families in *C. gigantea*

**Fig. 1:**
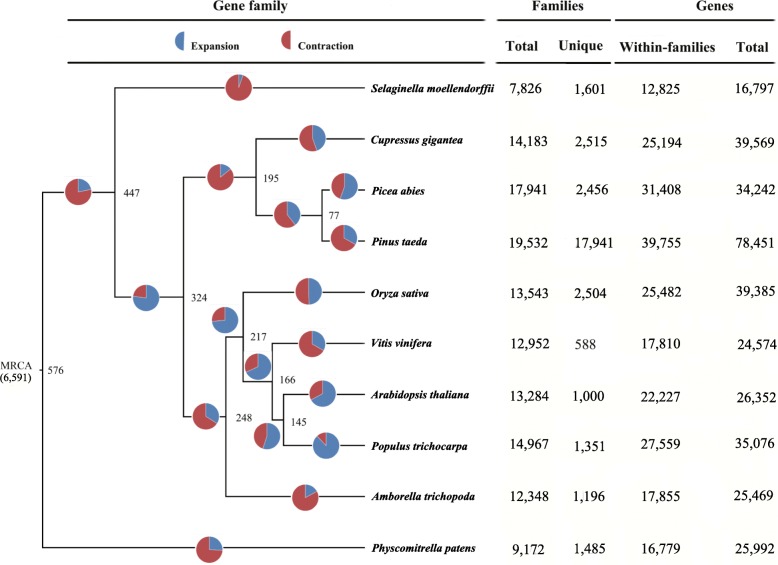
Phylogenetic relationships and number of gene families displaying expansion and contraction, respectively, among 10 plant species. Phylogenetic tree was constructed based on 4850 high-quality 1:1 single-copy orthologous genes identified by OrthoMCL, and moss species (*Physcomitrella patens*) was used as outgroup. Pie diagram on each branch of the tree represents the proportion of genes undergoing contraction (red) or expansion (blue) events. Number at root (6591) denotes the total number of gene families predicted in the most recent common ancestor (MRCA). The numerical values beside each node show the estimated divergent time of each node (myr). The amount of reconstructed gene families (the most left column), specific gene family identified (the second left column), genes within gene families (the second column from the right), and total genes (column in the most right) were presented

A core set of 41,373 genes belonging to 2412 orthologous gene families was shown to be shared by 10 species (*C. gigantea*; *Selaginella moellendorffii*; *Physcomitrella patens*; *Pinus taeda*; *Picea abies*; *A. trichopoda*; *A. thaliana*; *Populus trichocarpa*; *V. vinifera* and *O. sativa*) (Additional file 13: Figure S7). Species tree reconstruction based on 4850 single copy orthologous genes, divergence time, proportion of gene gain/loss and numbers of total and unique genes and gene families for each species are shown in Fig. 1. Based on 6591 gene families shown to be present in the most recent common ancestor (MRCA) of the 10 studied plant species, our estimate for the average rate of genomic turnover was 0.0011 gains and losses per gene per million years of evolution. We noted an overall increase in the number of gene families in all plant species examined since the MRCA around 576 million years ago. The *P. trichocarpa* lineage showed the largest number of gene family expansions (3592 families), and gene family contractions predominate for *S. moellendorffii*, *C. gigantean*, *P. taeda*, *V. vinifera*, *A. trichopoda and P. patens*. On the terminal lineage leading to *C. gigantean*, we inferred the gain of 1114 genes and the loss of 1383 genes since the split from the clade making by *P. taeda* and *P. abies*. We identified 2515 gene families containing 9223 genes only specific to *C. gigantea* (Additional file 13: Figure S7), and these genes were enriched in 587 GO categories (Table 1, Additional file 14: Table S7). The enriched categories included “root meristem growth” (GO: 0010449) and “regulation of plant organ morphogenesis” (GO: 1905421). Of particular interest were enrichments in several categories involved in interactions between organisms and environment, such as “systemic acquired resistance, salicylic acid mediated signaling pathway” (GO: 0009862) and “detection of external biotic stimulus” (GO: 0098581).

**Table 1:** Functional enrichment analysis of the *C. gigantea* gene family

Go term	Description	*P*-value	FDR
GO:0009862	systemic acquired resistance, salicylic acid mediated signaling pathway	3.61E-53	1.91E-49
GO:0010449	root meristem growth	2.07E-44	5.48E-41
GO:0009595	detection of biotic stimulus	1.32E-38	1.75E-35
GO:0098581	detection of external biotic stimulus	1.32E-38	1.75E-35
GO:0016045	detection of bacterium	5.01E-36	4.41E-33
GO:0098543	detection of other organism	5.01E-36	4.41E-33
GO:0010075	regulation of meristem growth	2.94E-35	2.22E-32
GO:0010082	regulation of root meristem growth	2.09E-31	1.38E-28
GO:0032412	regulation of ion transmembrane transporter activity	4.55E-29	2.67E-26
GO:0022898	regulation of transmembrane transporter activity	9.02E-29	4.76E-26
GO:0032409	regulation of transporter activity	2.22E-28	1.07E-25
GO:0052652	cyclic purine nucleotide metabolic process	5.50E-28	2.42E-25
GO:1900865	chloroplast RNA modification	2.10E-27	8.51E-25
GO:0009190	cyclic nucleotide biosynthetic process	3.05E-27	1.15E-24
GO:0006171	cAMP biosynthetic process	5.02E-27	1.77E-24
GO:1905421	regulation of plant organ morphogenesis	5.34E-26	1.76E-23
GO:0044426	cell wall part	2.98E-25	9.27E-23
GO:0044462	external encapsulating structure part	9.70E-25	2.84E-22
GO:0046058	cAMP metabolic process	1.08E-24	2.93E-22
GO:0048226	Casparian strip	1.11E-24	2.93E-22

### Discovery of gene families related to resistance in *C. gigantea*

**Fig. 2:**
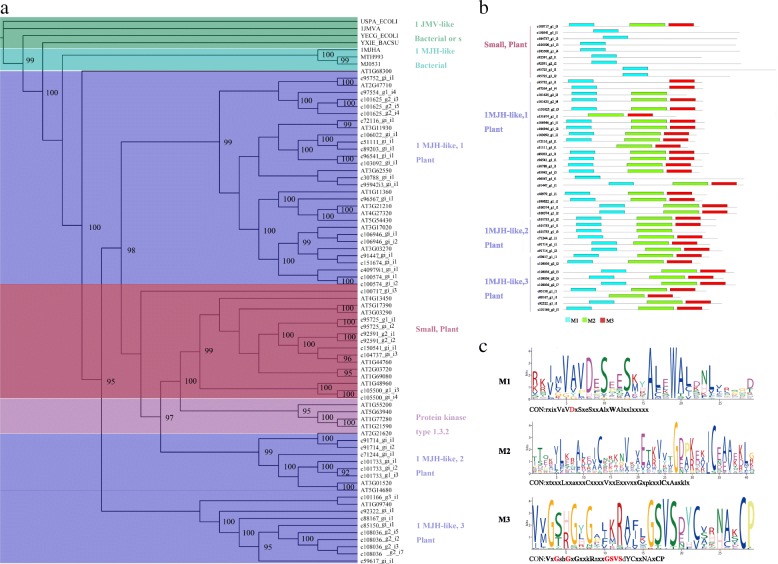
45 USPA-like domain sequences were identified in the *C. gigantea* transcriptome. **a** Phylogenetic tree of the USPA-like domain from *C. gigantea* transcriptome and characterized representative USPA-like from a broad range of plant and bacterial lineages. **b** MEME motif distribution of the 45 USPA-like domain sequences. **c** Conserved motifs in USPA-like domain and their consensus sequences. CON indicates consensus sequence. If the bits value of amino acid at this position was smaller than 1, it was represented with x; 2 > bits ≥1, with lowercase; 3 > bits ≥2, with capital letter; bits ≥3, with bold capital

In our study, 45 sequences with USPA-like domain were identified in the *C. gigantea* transcriptome. A phylogenetic tree was constructed from multiple sequence alignment of USPA-like domains with a total of 77 sequences (45 *C. gigantea*, 25 *Arabidopsis* and 7 bacteria). Within the ML tree (Fig. 2a), the sequences clearly fell into several distinct groups of USPA-like domain consistent with the previously established nomenclature [30]. All sequences from *C. gigantea* with putative USPA like-domains were classified into two groups: 1MJH-like_Plant and Small_Plant categories. The sequences of the 1MJH-like group from *C. gigantea* were further subdivided into three subgroups, 1MJH-like1, 1MJH-like2 and 1MJH-like3, respectively. We found that 80% of USPA-like domain sequences from *C. gigantea* corresponded to the 1MJH-like group, yet relatively few corresponding loci were detected for the Small_Plant group category. All USPA-like sequences from *C. gigantea* unigenes and *A. thaliana* were closely related to the group formed by 1MJH-like USPA genes from bacteria, which is consistent with a previous study [31].

To explore evolutionary divergence of the USPA-like domain among sequences collected here, we performed motif analyses using the MEME program. MEME analysis identified 3 motifs in the USPA-like domain, classified from the N terminus to the C terminus as M1, M2, and M3. (Fig. 2b). The alignment of the USPA-like domain with all known secondary structure elements and conserved residues is showed in Additional file 15: Figure S8. We found motif M1 is shared across all groups and almost all members of each group with motifs M2 and M3 were also shared across most groups, with the exception of the Small_Plant group. As much as 64% of the identified unigenes in *C. gigantea* contained 3 motifs, and for the remaining 36% 2 motifs (16%) to only 1 motif (20%) were found. Seven out of the eight most conserved residues (D13, V41, G127, G130, G140, S141, V142, and T143) within the USPA structure for 1MJH and ATP binding were identified for the USPA-like unigenes from *C. gigantea.*

**Fig. 3:**
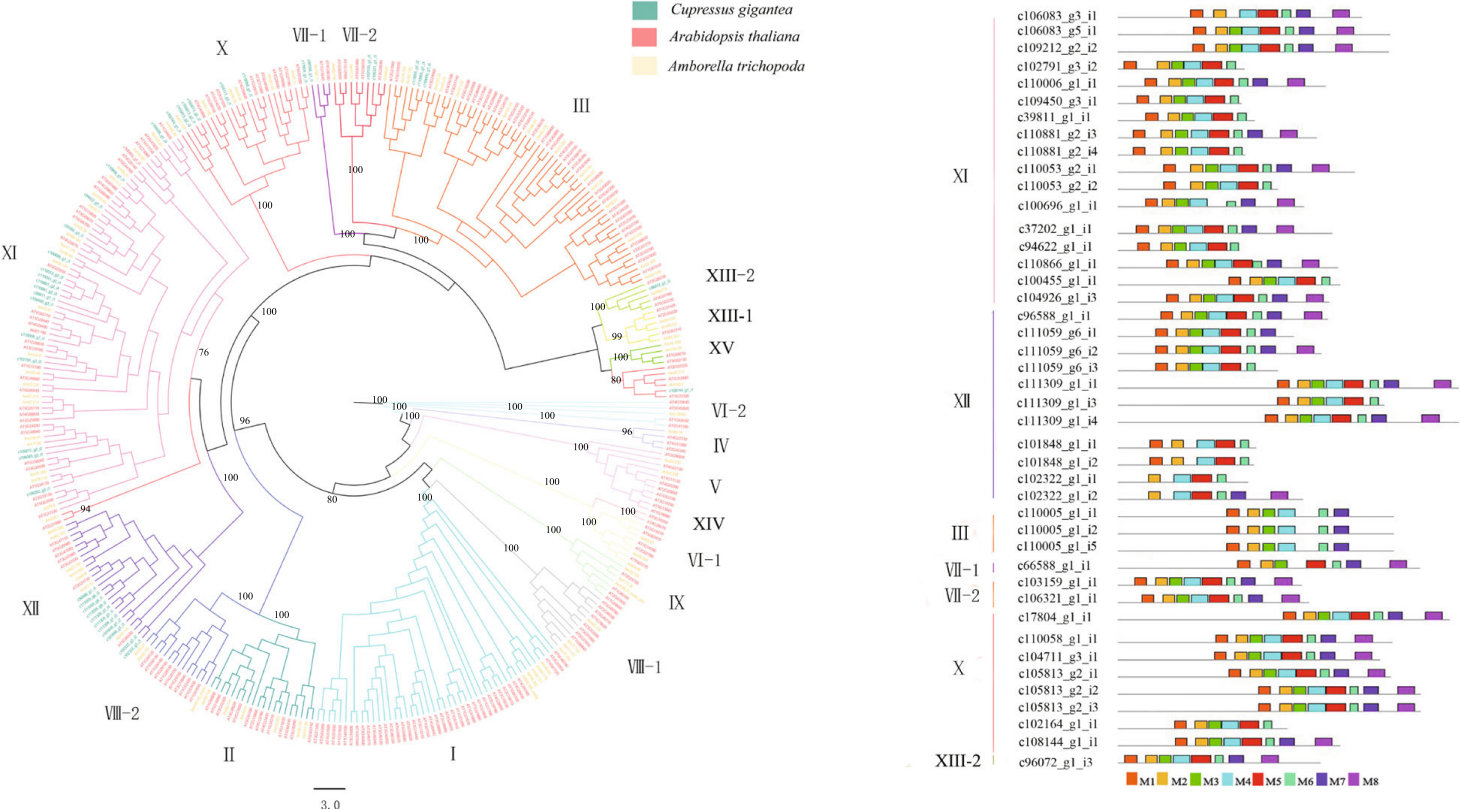
Phylogenetic tree with 350 LRR-RLK unigenes from the *C. gigantea* transcriptome, *Amborella trichopoda* and *Arabidopsis thaliana* genomes, with 35 LRR-RLK motif unigenes from the *C. gigantea* transcriptome. **a** ML tree of the 35 USPA-like domain from *C. gigantea* transcriptome and 94 LRR-RLK genes from the *Amborella trichopoda* genome and 213 LRR-RLK genes from *Arabidopsis thaliana*. Color legend correspond to the species of gene origination, and the colors of the branches correspond to different sub-families. **b** MEME motif distribution of the 35 USPA-like domain sequences

We identified 43 LRR-RLK unigenes from *C. gigantea* after confirming the presence of the extracellular domain (ECD), intracellular kinase domain (KD), and transmembrane (TM) domain. Subsequently, we combined 94 LRR-RLK genes from *A. trichopoda* and 213 from *A. thaliana* for further analysis, as for these two representative angiosperm we could collect more background resources, such as phylogeny, classification and functional characterization of LRR-RLK. In total, 350 LRR-RLK sequences were used to construct the ML phylogenetic tree. The *C. gigantea* sequences that clustered together with known members of *A. thaliana* LRR-RLK were assigned to the corresponding group by referring to a previous study [32]. As shown in the tree (Fig. 3a), the LRR-RLK genes are divided into 19 different subfamilies, of which subfamily X is separated into 3 groups. Most subfamilies were highly supported with bootstrap values ranging between 94 and 100%, except for subfamily XI with only 76%. Of the 19 LRR-RLK subfamilies, only subfamily XIV did not include *C. gigantea* and *A. trichopoda*; subfamilies I, II, IV, V, VI-1, VI-2, VII-1, VII-2, IX, XII-1 and XV did not include *C. gigantea* and all the other subfamilies III, VII-1, VII-2, X, XI, XII, XIII-2 included LRR-RLK sequences from all three species. The number of unigenes from *C. gigantea* was unevenly distributed across subfamilies. We found subfamilies XI and XII had the largest members of unigenes, and subfamilies VII-1 and XIII-2 contained only one unigene.

Although the KD domain is relatively well conserved, it can be divided into 12 smaller subdomains useful for elucidating evolutionary divergence [33, 34]. In our study, 43 sequences from *C. gigantea* classified as LRR-RLK were identified with the MEME program. According to the position of the kinase domain and conserved amino acid residues, eight motifs (M1-M8) are shown in Fig. 3b and Additional file 16: Figure S9, and which contain 11 subdomains in total but without the X subdomain which is the most poorly conserved subdomain and its function is also unknown [33]. M2 and M6 motifs are shared across all LRR-RLK proteins identified in *C. gigantea*. Motifs M1, M5, M6, M7 and M8 correspond to conserved subdomains I & II, VIb & VII, VII, IX, and XI, respectively. These motifs are shared by almost all subfamilies except for motifs M5 and M8 that are not shared by any members of subfamily III. Meanwhile, two less conserved subdomains were also found. Motifs M3 and M4 correspond to subdomains V and VIa. These motifs are shared by almost all LRR-RLK genes. In addition, motif M2 corresponds to two subdomains, with conserved subdomain III and less conserved subdomain IV. The motif is shared by all subfamilies and all members of each subfamily.

**Fig. 4:**
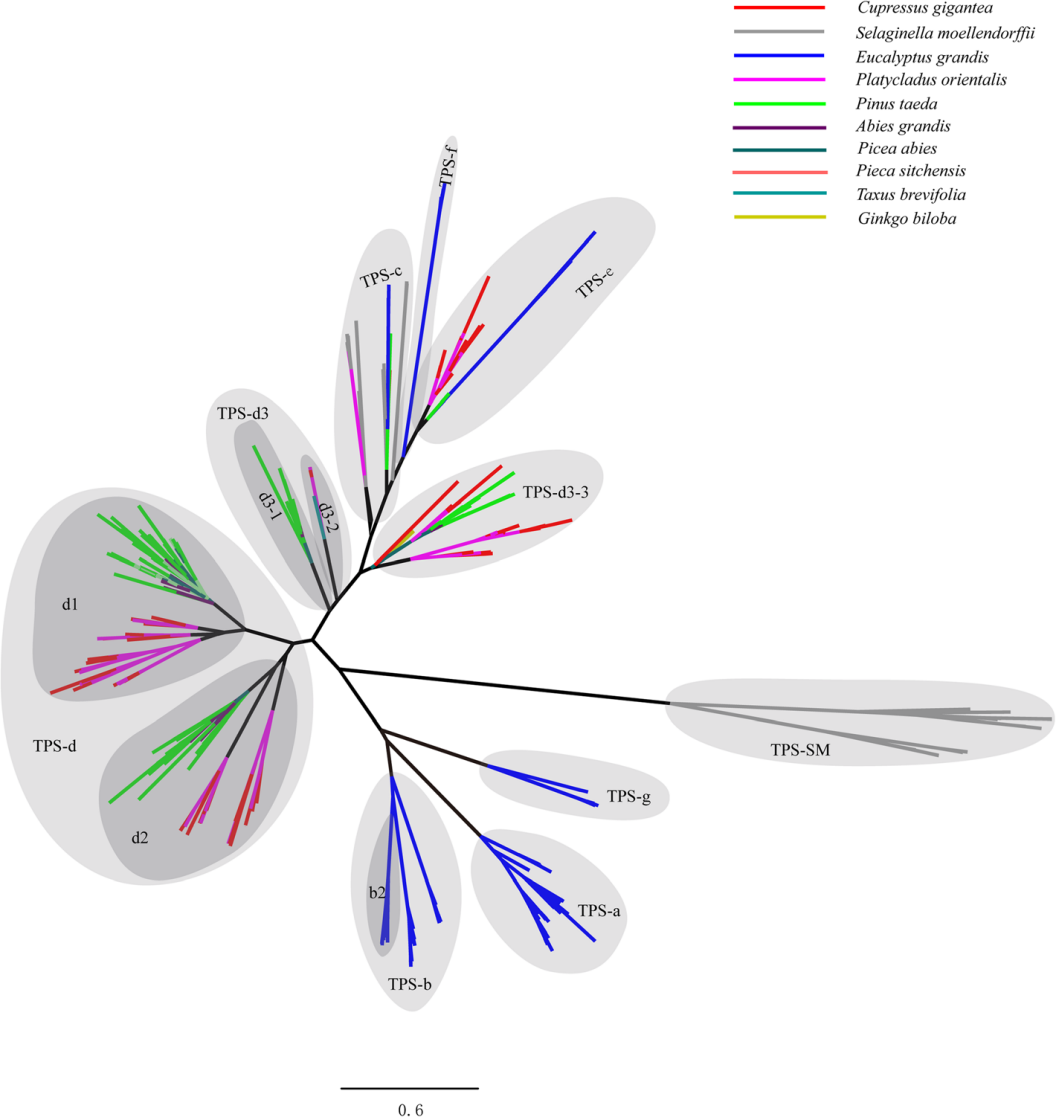
ML tree with 426 TPS sequences obtained from the *C. gigantea* transcriptome comparing to nine other species genomes

A total of 426 TPS sequences obtained from *C. gigantea*, *Platycladus orientalis*, *P. taeda*, *Abies grandis*, *P. abies*, *P. sitchensis*, *Taxus brevifolia, S. moellendorffii*, *E. grandis* and *Ginkgo biloba* were used for phylogenetic analyses. The topology of the ML phylogenetic tree allowed us to divide TPS into 8 subfamilies following TPS-a, TPS-b, TPS-c, TPS-d, TPS-e, TPS-f, TPS-g, TPS-SM according to previous evolutionary analyses [35–39]. In the present study, we substantially expanded these analyses to include 121 new TPS unigenes from *C. gigantea*. (Fig. 4). Unlike previous studies, the TPS-d3 subfamily was divided into the three groups, TPS-d3–1, TPS-d3–2, and TPS-d3–3, respectively. It was previously reported that the TPS-d3 subfamily is gymnosperm-specific, and mainly contains diterpene synthases and several sesquiterpene synthases [38]. Group TPS-d3–1 contains sesquiterpene synthases, while the other two groups primarily contain diterpene synthases, TPS-d3–2 contained mainly taxadiene synthase from *Taxus* and TPS-d3–3 levopimaradiene synthase from *G. biloba*. *C. gigantea* TPS unigenes were mainly distributed across TPS-d1, TPS-d2 and TPS-e subfamilies with 30, 31, 29 unigenes, respectively, while the TPS-1 subfamily only possessed one TPS unigene from *C. gigantea*. The TPS-d3–2 subfamily also showed a very low number of unigenes from *C. gigantea*, with only two unigenes.

Plant TPSs can be classified into two groups, class I and class II, with a DDxxD motif involved in divalent metal binding and considered a characteristic feature of class I TPS [40, 41]. In our study, the DDxxD motif in *C. gigantea* varied, occurring as DD (I/L/F/T/C) (Y/F) (D/Y/E) starting at the protein sequence position after about 480 amino acids (Additional file 17: Figure S10). Among the TPS unigenes from *C. gigantea*, motif DDxxD is shared by almost all subfamilies, with the exception of the TPS-c subfamily. In addition, a conserved RxR motif located upstream of the DDxxD motif was also found by using the MEME program. The RxR motif varied in the TPS-e subfamily occurring as RxK. The RxR motif produced 69 hits while the DDxxD motif identified 77 sites according to their position and conserved amino acid residues within the 121 TPS unigenes from *C. gigantea*, for which 50 TPS unigenes contained RxR and DDxxD motifs. Furthermore, RxK and DDxxD motifs were distributed across 16 unigenes of TPS-e subfamily in *C. gigantea.*

### Validation of the presence and the potentially functional divergence among the individual gene members

Reverse Transcription PCRs (RT-PCRs) successfully amplified targets sequences with 35 out of 36 pairs of primers designed for amplification of gene members from USPA (primer pairs designed to amplify target from 9 gene members), LRR-RLK (13) and TPS (14) gene families. PCR with DNA as template amplified the target sequence with the only primer pair (c106946_g1_i2) which failed to amplify target in RT-PCR. Successful amplification indicated the high fidelity of our transcriptome gene assembly. Further real-time quantitative reverse transcription PCR (qRT-PCR) revealed significantly different gene expression among gene members from the same family, suggestive of the potentially functional divergence among the gene members. See Additional file 18: Figure S11 and Additional file 19: Figure S12 for gel electrophoresis from RT-PCR and PCR and results of differential gene expression tests.

## Discussion

Cypresses are endemic trees or shrubs prone to disjunctive distributions in temperate regions throughout the northern hemisphere [5]. Because of its isolation, *C. gigantea* has a narrow and scattered distribution located only in the high altitude region of the Tibetan plateau in the southwest of China and its populations sizes are small. Due to low reproductive output, difficulty of seed germination, and the severe environmental factors associated with high elevation *C. gigantea* remains endangered [7]. Considering the urgent need for *C. gigantea* conservation and functional characterization of the species’ adaptive potential, its global transcriptome characterization could provide the basic genomic information for future assessment of the species’ genetic variation at the molecular level. In the present study, circa 14.41 million high quality reads were assembled into 101,092 unigenes, with an average sequence length of 1029 bp. The transcriptome data of *C. gigantea* was compared to those of other conifers whose genomic data were recently released (Additional file 20: Table S8).

Gene family membership may be reduced due to incomplete expression of the proteome. However, the high quality of the transcriptome data still enabled us to discover and annotate genes associated with fundamental evolutionary processes. Our results indicated that there are 2515 unique gene families (containing 9223 genes) unique to the *C. gigantea* lineage following its divergence from the most recent common ancestor shared with any other taxon. Functional annotation of 3804 unigenes demonstrated that they are mainly enriched in functional categories involved in biological processes, including systemic acquired resistance and the salicylic acid mediated signaling pathway (GO:0009862) and the detection of biotic and external biotic stimuli (GO:0009595, GO:0098581).

Universal stress protein A (USPA) was proposed to have two domain types, with 1MJH involved in ATP-binding and 1JMVA without ATP-binding potential. Many studies have shown that the USPA-like domain is derived from a 1MJH-like ancestor in plants such as *A. thaliana* [30], *Catharanthus roseus* [31] and animals such as urochordates as well as all Cnidaria and Lophotrochoza [42]. In the present study, the phylogenetic and structural analyses also indicated that USPA domains derived from a 1MJH-like ancestor for *C. gigantea.* We found that all *C. gigantea* sequences were more closely related to the bacterial 1MJH than to 1JMVA with strong bootstrap support in the phylogenetic tree. Moreover, we obtained the conserved motifs of the USPA-like domain in *C. gigantea* through MEME analysis which is basically consistent with the annotated structure of 1MJH except for the alpha 3 block. The eight residues (D13, V41, G127, G130, G140, S141, V142, and T143) in the USPA structure of 1MJH needed for ATP binding were also identified in the alignment of USPA-like domains, yet the V41 located at beta 2 block of 1MJH that binds adenine was not found in the motif. It may be that the beta 2, alpha 2, beta 3, and alpha 3 blocks are less conserved than other motifs. In addition, it is possible that such genetic variation was further shaped during the evolution of *C. gigantea*.

Leucine-rich repeat receptor-like protein kinases (LRR-RLK) genes were classified into 19 sub-families according to our phylogenetic analysis, which is consistent with previous studies [43]. However, LRR-RLK genes from *C. gigantea* were divided into 7 sub-families, i.e. III, VII-1, VII-2, X, XI, XII, XIII-2 sub-families, respectively. Considering that we used transcriptomes, the information of genes may be incomplete due to transcripts that were not expressed in our sample and thus, information could have been missed for other sub-families in *C. gigantea*. Twelve kinase domain sub-families have already been recognized and also implicated in playing essential roles in enzyme function [33]. In the present study, we identified 8 motifs containing 11 sub-families through MEME motif analysis. M2 and M6 motifs are shared by all LRR-RLK proteins identified in *C. gigantea*. These common motifs indicate functional similarities related to kinase activity. The M2 motif corresponds to the sub-family III with conserved residues and sub-family IV with less conserved residues, and M6 corresponds to sub-family VII with conserved residues. Sub-families III and VII contain invariant residues that are crucial for maintaining kinase activity and peptide substrate recognition [33, 34]. Sub-family IV contains no invariant or nearly no invariant residues and therefore does not appear to be directly involved in catalysis or substrate recognition [33].

Terpenoids are compounds widely found in plants, and they are the main components of some resins, which provide resistance to biotic challenges such as disease causing agents. Conifers may also have a large and diverse terpene synthase (TPS) gene family given the diversity of TPS genes that have been characterized in other studied species [44]. The 113 and 106 putative functional TPS genes identified in *E. grandis* and *E. globulus*, respectively, represent approximately four times as many as in *A. thaliana* (40 putative functional genes) [25, 45]. In our study, we discovered 121 such unigenes in the *C. gigantea* transcriptome. Phylogenetic analyses of TPS unigenes recognized eight major sub-families, designated TPS-a through TPS-g and TPS-SM. The TPS-d3 sub-family was further divided into three groups that were renamed d3–1, d3–2, d3–3 according to various terpene molecules. More convincingly, the conserved and short amino acid sequence DDXXD, implicated in catalytic function with Mg^2+^ binding, was also identified. Two conserved motifs in TPS, namely RXR and DDXXD, are separated by a short region of 35 amino acids. The two motifs are thought to direct the diphosphate ion away from the carbocation upon cleavage of the preny1 diphosphate substrate [46].

## Conclusion

This study provides the first comprehensive transcriptome analysis of *C. gigantea*. In total, 101,092 unigenes with high sequence quality were obtained and were functionally classified based on BLASTx searches across multiple databases. Putative universal stress proteins (USPs), leucine-rich repeat receptor-like protein kinases (LRR-RLKs) and terpene synthase (TPS) genes found in *C. gigantea* are known to be involved in many different biotic and abiotic stress responses. We described a set of 2515 specific gene families containing 9223 genes for *C. gigantea* and demonstrated their usefulness for phylogenetic reconstruction. These unigenes and their analyses will likely form the foundation for future genetic analyses in *C. gigantea*, and we strongly believe that this public transcriptome database will serve as an important information platform to help us understand the genetic underpinnings of stress adaptation in *C. gigantea* and other closely related species. The present study demonstrates large-scale transcriptome sequencing and in-depth analyses to be a valuable means to resolve the genomics of extremophile adaptation in species with prohibitive genome size.

## Materials and methods

### Plant material and RNA isolation

Microstrobili (male pollen cones), female strobili, terminal buds, biennial leaves, and cambium tissues originated from five adults of *C. gigantea* growing in the Giant Cypress Nature Reserve (Nyingchi, Tibet, China). The plant material was collected in May 2014 (active stage of physiological activity), with permission from the local forestry bureau. All sampled tissues were immediately flash frozen in liquid nitrogen and stored at − 80 °C until RNA extraction. Total RNA was isolated from each tissue using a RNeasy Plant Mini Kit (Qiagen, Hilden, Germany). In total, 25 RNA samples were prepared, representing the five tissue types from each of the five sampled trees. RNA was quantified and quality-checked for each sample; RNA was then pooled in one tube in equal quantities for RNA-Seq analysis.

### cDNA library preparation and Illumina sequencing

The mRNA library was constructed according to the manufacturer’s instructions using the mRNA-Seq Sample Preparation Kit (Illumina, Inc., San Diego, CA, USA). The poly-(A) mRNA was isolated from the total RNA samples using magnetic oligo (dT) beads. To avoid priming bias, the mRNA was fragmented using an RNA fragmentation kit (Ambion, Austin, TX, USA) before cDNA synthesis. The cleaved RNA fragments were transcribed into first-strand cDNA using reverse transcriptase (Invitrogen, Carlsbad, CA, USA) and random hexamer primers, followed by second-strand cDNA synthesis using DNA polymerase I (New England BioLabs (NEB), Ipswich, MA, USA) and RNaseH (Invitrogen). Short fragments were purified with the QiaQuick PCR extraction kit. Thereafter, the short fragments were connected with sequencing adapters. Following agarose gel electrophoresis, 300-600 bp long fragments were selected for PCR amplification as templates. Finally, the library was sequenced using Illumina HiSeq™ 2500 and sequences were deposited in the GeneBank Short Read Archive (Accession SRX2996533).

### De novo transcriptome assembly

Raw reads were filtered to obtain high-quality clean reads by removing adaptor sequences, duplicated sequences and ambiguous reads (reads with unknown nucleotides “N” > 5%) using Trimmomatic (version 0.36) software [26] with default parameter settings; bases with Phred score < 20 were trimmed. Based on the quality check, the last two base pairs from each read were removed in order to minimize the overall sequencing error. Additional file 1: Figure S1 and Additional file 2: Figure S2 show quality assessment using FastQC [47]. Subsequently, *do novo* assembly of the transcriptome was carried out with the short read assembly program Trinity using default parameters [27].

The unigenes generated by Trinity were annotated using the Non-Redundant protein database from NCBI (Nr), KOG [28] of the Cluster of Orthologous Groups for eukaryotic complete genomes (COG) database [48], and Gene ontology (GO) protein database [49], with a cut-off E-value of 1.00E-5. For Nr annotation, we used the Blast2GO program (version 3.1) to obtain the GO annotation of unigenes [50]. After obtaining the GO annotation for each unigene, we used the WEGO software to perform GO functional classification for all unigenes [51]. Unigenes were associated to metabolic pathway constructed by the Kyoto Encylopedia of Genes and Genomes (KEGG) [52], and this was done using a Blastall search [53] against the KEGG database.

### Gene expansion test

Protein coding sequences from the *C. gigantea* transcriptome and nine other plants including one lycophyte species (*Selaginella moellendorffii,* one bryospida species (*Physcomitrella patens*), two gymnosperm species (*Pinus taeda* and *Picea abies*) and five angiosperm species (*Amborella trichopoda*, *Arabidopsis thaliana*, *Populus trichocarpa*, *Vitis vinifera* and *Oryza sativa*) were obtained from PLAZA database (http://bioinformatics.psb.ugent.be/plaza/) [54]. Alternatively, spliced and redundant sequences were removed and only the longest isoforms were retained. Filtered sequenced were first grouped with all-by-all comparisons using blastp, and significant hits (E-value < 10^− 5^) were clustered into gene families with the Markov cluster algorithm (MCL) in the OrthoMCL package (version 2.0.9) [55]. The coding sequences for each gene were individually retrieved and aligned using MAFFT (version 7.335) [56]. The alignments were further concatenated to construct a gene tree for each plant species. We finally constructed the phylogenetic tree between the cypress tree and nine other plant species using RAxML package (version 8.1.24) [57] under the following parameter settings: “-f a -x 12345 -p 12345 -# 100 -m PROTGAMMAILGX -T 4”. To track the phylogenetic history of gene families and identify expansions, we used the program CAFÉ (version 3.1) following the parsimony rule to reconstruct ancestral states [58]. Functional annotation of specific gene families in *C. gigantea* was performed using the Trinotate program [59]. Further Gene Ontology (GO) enrichment analysis was conducted by clusterProfile 3.8.1 package (http://bioconductor.org/packages/clusterProfiler/) with false discovery rate (FDR) corrections.

### Gene families and protein structure analysis

Unigenes with universal stress protein USPA-like domains were identified within the *C. gigantea* transcriptome by querying the PLAZA database (https://bioinformatics.psb.ugent.be/plaza/versions/gymno-plaza/) [54] with the online analytical tool TRAPID (http://bioinformatics.psb.ugent.be/webtools/trapid/) [60]. For comparison, the USPA-like sequences were also retrieved from *Arabidopsis thaliana* by mining the PLAZA database [54]. To track the evolutionary ancestor of the USPA sequences from *C. gigantea*, a collection of bacterial USPA proteins was obtained from a previous study [30] containing two proposed crystal structures of USPs, one from *Mechanococcus jannaschii* (1MJH) with binding ATP and the other from *Haemophilus influenza* (1JMV) without binding ATP. Putative USPs sequences were examined using the CDD (conserved Domain Database) (https://www.ncbi.nlm.nih.gov/cdd) and Pfam databases (PF00582) (http://pfam.xfam.org/) to further verify the presence of conserved USPA-like domains. Identical or defective sequences were identified and eliminated by manual inspection in BioEdit [61]. Unigenes with USPA-like domain from *C. gigantea*, *Arabidopsis thaliana* and bacteria were retrieved and analyzed.

Putative LRR-RLK unigenes of *C. gigantea* were identified within the *C. gigantea* transcriptome assembly using TRAPID (see above). For all obtained LRR-RLK unigenes, we employed CDD (https://www.ncbi.nlm.nih.gov/cdd) to confirm the presence of ECD and KD domains, and TM domains were predicted by querying the TMHMM website (http://www.cbs.dtu.dk/services/TMHMM/) with default parameters of version 2.0. Unigenes not belonging to the LRR-RLK family were rejected. In addition, more than 200 LRR-RLK genes have been retrieved from previous studies where LRR-RLK members were identified in the whole genome sequences of *Arabidopsis thaliana* [62] and *Amborella trichopoda* [43]. *A. thaliana* and *A. trichopoda* were chosen as the representatives as we saw they have genomic and functional resources in high quality and also *A. trichopoda*, one basal angiosperm species, was a good reference for our present study on conifer.

We identified unigenes in the *C. gigantea* transcriptome that showed significant similarities to known terpene synthase (TPS) genes, again using TRAPID. And, TPS family members were identified for *Pinus taeda*, *Ginkgo biloba* and *Selaginella moellendorffii* which with whole genome sequences available, by mining the PLAZA database (http://bioinformatics.psb.ugent.be/plaza/) [54]. TPS members from the other five conifers (*Platycladus orientalis* [39], *Abies grandis* [37], *Picea abies* [37], *Pieca sitchensis* [37], *Taxus brevifolia* [37]), and one angiosperm (*Eucalyptus grandis* [25]) were also retrieved and analyzed. A preliminary list of hits was created and redundancies were removed. All of the obtained TPS sequences were retained and examined by querying CDD database at NCBI.

Multiple sequence alignments were conducted for all amino acid sequences originating from USPs, LRR-RLKs and TPS families, respectively, using MAFFT version 7.335 [56] following default settings. The aligned sequences were visualized and manually refined with Jalview version 2.0 [63]. Alignments were further filtered using trimAL (version1.3) with gappyout method [64]. Maximum likelihood trees were constructed with Phyml version 3.0 [65] using JTT amino acid substitution model, and the branch support was estimated with approximate likelihood tests and 1000 bootstrap replicates. Phylogenetic trees were visualized and annotated using FigTree v1.4.2 [66]. In addition, we identified conserved motifs for *C. gigantea* unigenes with Multiple Expectation Maximization for Motif Elicitation (MEME) v.4.11.3 [67]

### Real-time quantitative reverse transcription PCR (qRT-PCR)

To validate the presence and the potentially functional divergence among the individual gene members from the same gene family we reconstructed here, qRT-PCR was further executed. Primers were designed with Primer Premier 5.0 software (available from frodo.wi.mit.edu/cgi-bin/primer5/primer5_www.cgi). In total, 35 primer pairs were successfully designed for amplification of 35 genes from 15 groups/subfamilies of the three gene families (USPA, LRR-RLK and TPS). Primer sequences were provided in Additional file 21: Table S9. qRT-PCR was conducted on LightCycler® 96 Thermocycler (Roche, Mannheim, Germany) using SYBR Premix Ex Taq (TaKaRa, Toyoto, Japan). Reactions were prepared in a total volume of 20 μl (containing 1 μl of template, 10 μl of 2 × SYBR Premix, 0.8 μl of each specific primer and 8.4 μl of ddH_2_O). The reactions conditions were performed as following: 5 min at 95 °C, 40 cycles of 95 °C for 15 s, 60 °C for 20 s and 72 °C for 15 s. Baseline and threshold cycles (Ct) were automatically determined using the LightCycler® 96 software version SW 1.1 (Roche, Mannheim, Germany). Relative gene expression with respect to internal reference gene, Actin 7, was determined with 2^-(Δ*Ct*)^ methods (ΔCt = Ct of the target - Ct of the reference) [68]. Kruskal-Wallis H test was used to test significance of differences on gene expression among different groups/subfamilies. Kruskal-Wallis H test was implemented with ‘kruskal’ function from R package ‘agricolae’ [69]. Before qRT-PCR, reverse transcription polymerase chain reaction (RT-PCR) and PCR, with RNA and DNA as template, respectively, were executed to validate the presence of the assembled gene members. RNA and DNA from leaves of two individual trees were used as templates for these RT-PCR and PCR amplification. We applied 2–3 replicates for one specific amplification.

## Additional files

Additional file 1: **Figure S1.** Assessment of reads by FastQC before quality control. a) Quality of raw-reads per base. The central red line is the median base quality, the yellow box represents the interquartile range (25–75%), the upper and lower whiskers represent the 10 and 90% points, respectively, and the blue line represents the mean base quality. b) Distribution of raw-reads per base. c) The mean sequence quality scores over all reads. d) Distribution of sequence lengths over all sequences. (PDF 24825 kb)

Additional file 2: **Figure S2.** Assessment of reads using FastQC after quality control. a) Quality of reads per base after adaptive window trimming using a quality average threshold of 20 and a minimum length threshold of 20. The central red line is the median value, the yellow box represents the interquartile range (25–75%), the upper and lower whiskers represent the 10 and 90% points, respectively, and the blue line represents the mean base quality. b) Sequence content across all bases. c) Distribution of the mean quality scores over all sequenced reads. d) Length distributions of all sequenced reads. (PDF 23882 kb)

Additional file 3: **Table S1.** Summary of assembled contigs, scaffolds and unigenes properties for the *C. gigantea* unigenes. (DOCX 15 kb)

Additional file 4: **Figure S3.** Length distributions of all unigenes for *C. gigantea*. (PDF 596 kb)

Additional file 5: **Table S2.** Summary of database matches (specific values) for *C. gigantea* unigenes. (DOCX 15 kb)

Additional file 6: **Table S3.** Database matches (full results) for *C. gigantea* unigenes. (XLSX 1763 kb)

Additional file 7: **Table S4.** BLASTX hits for all unigenes in the Nr database. (XLSX 4233 kb)

Additional file 8: **Table S5.** Swiss-Prot annotations for all unigenes. (XLSX 2954 kb)

Additional file 9: **Figure S4.** GO annotation of *C. gigantea* unigenes. (PDF 2723 kb)

Additional file 10: **Figure S5.** KOG classification of *C. gigantea* unigenes. (PDF 1375 kb)

Additional file 11: **Table S6.** KEGG pathway annotations for the assembled unigenes. (XLSX 173 kb)

Additional file 12: **Figure S6.** Metabolic pathway of the Terpenoid bakcone biosynthesis for the unigenes identified in *C. gigantea*. Each box represents the substance involved in each section of the pathway. The red boxes represent substances assigned at least one unigene. (PDF 530 kb)

Additional file 13: **Figure S7.** Shared and unique gene families among *C. gigantea* and nine other plant species. (PDF 9 kb)

Additional file 14: **Table S7.** Functional enrichment analysis of the specific gene families from *C. gigantea*. The full list of Gene Ontology (GO) enriched functional categories are shown. (XLSX 108 kb)

Additional file 15: **Figure S8.** Multiple sequence alignment (MSA) of the USPA-like domains of the putative USPA protein. The annotation was done based on conserved features of 1MJH secondary structure (five β strands and four α helices). (PDF 6074 kb)

Additional file 16: **Figure S9.** Conserved motifs in LRR-RLK domain from *C. gigantea* transcriptome and their consensus sequences. Conserved motifs for the LRR-RLK domain from *C. gigantea* transcriptome and their consensus sequences. ‘CON’ indicates the consensus sequence. If the bits value of amino acid at this position is smaller than 1, it is represented with x; 2 > bits ≥1, with lowercase; 3 > bits ≥2, with capital letter; bits ≥3, with bold capital. (PDF 942 kb)

Additional file 17: **Figure S10.** Alignment of the conserved RxR and DDxxD motifs and motifs variation among 121 TPS sequences from *C. gigantea* transcriptome, with their corresponding consensus sequences. Multiple sequence alignment of the TPS domain sequences from *C. gigantea* transcriptome. Conserved motifs for the TPS domain from *C. gigantea* transcriptome and their consensus sequences. ‘CON’ indicates consensus sequence. If the bits value of amino acid at this position was smaller than 1, it was represented with x; 2 > bits ≥1, with lowercase; 3 > bits ≥2, with capital letter; bits ≥3, with bold capital. (PDF 7804 kb)

Additional file 18: **Figure S11.** Gel electrophoresis of 39 primers for real-time PCR with cDNA as template (a and b) and a subset (c and d) primers sets using cDNA as template for qRT-PCR. PCR was performed (d) with one primer pair failed to amplify target in RT-PCR using DNA as template (two columns on the most right side). The numbers on top of each plot (a, b, c and d) indicate the code of primer used for PCR amplification, refer to Supplementary S Table 9 for details of each primer pair. (PDF 870 kb)

Additional file 19: **Figure S12.** Test of differential gene expresssion among selected genes from three gene families (USPA, LRR-RLK and TPS) based on quantitative real-time RT-PCR (qRT-PCR). Relative Actin gene expression and result of test on differential gene expression among different genes were shown. (PDF 386 kb)

Additional file 20: **Table S8.** Characteristics of the transcriptome assemblies from related conifer species and re-analysis results from TRAPID. (XLSX 19 kb)

Additional file 21: **Table S9.** Sequences of primers for real-time PCR. (DOCX 23 kb)

## Abbreviations

ECD: Extracellular
KD: Kinase domain
LRR-RLKs: Leucine-rich repeat receptor-like protein kinases
TM: Transmembrane
TPS: Terpene synthase
USPA: Universal stress protein A
