# Supplementary figures and images for: In-depth transcriptome characterization uncovers distinct gene family expansions for Cupressus gigantea important to this long-lived species’ adaptability to environmental cues
Source: BMC Genomics. 2019 Mar 13;20:213. doi: 10.1186/s12864-019-5584-6 (PMC6417167; doi:10.1186/s12864-019-5584-6)

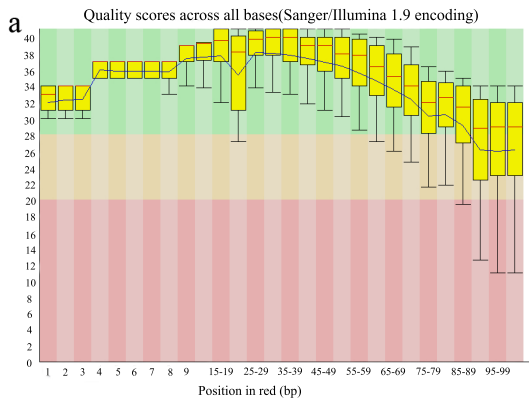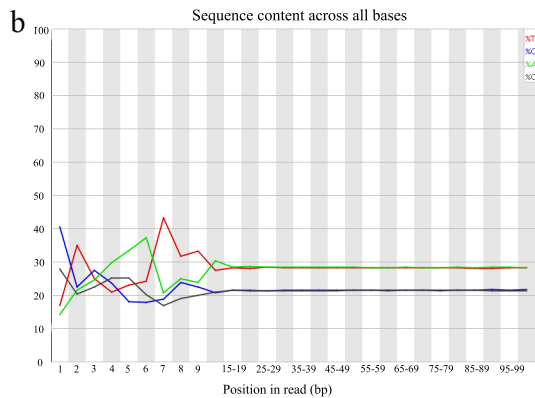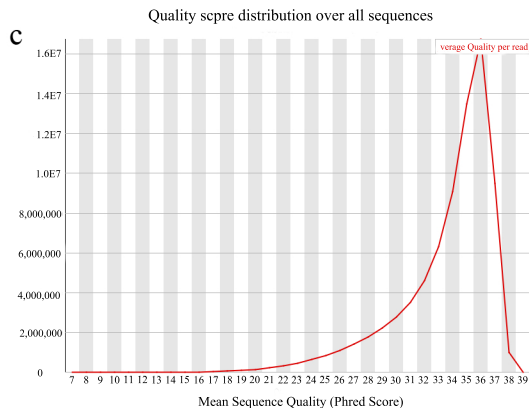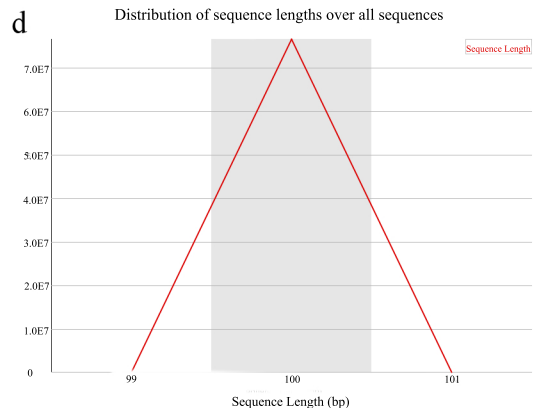

Supplement: Supplementary file 1 — Figure S1. Assessment of reads by FastQC before quality control. a) Quality of raw-reads per base. The central red line is the median base quality, the yellow box represents the interquartile range (25–75%), the upper and lower whiskers represent the 10 and 90% points, respectively, and the blue line represents the mean base quality. b) Distribution of raw-reads per base. c) The mean sequence quality scores over all reads. d) Distribution of sequence lengths over all sequences. (PDF 24825 kb) [file 12864_2019_5584_MOESM1_ESM.pdf]

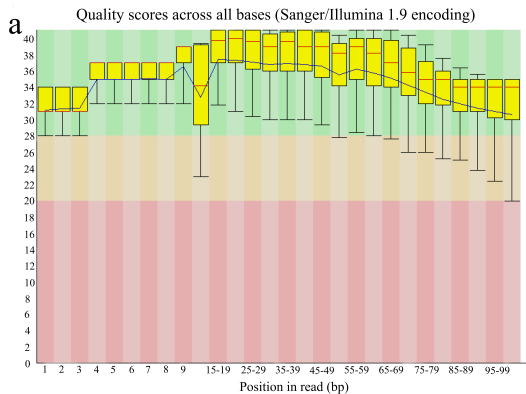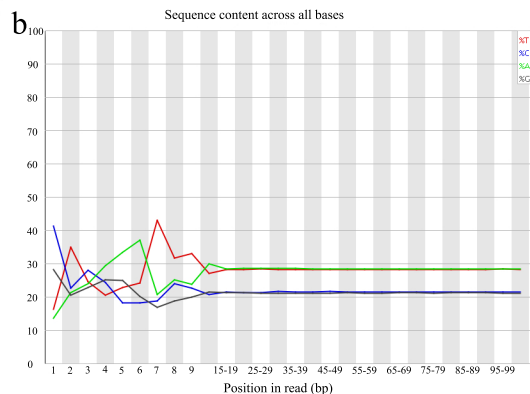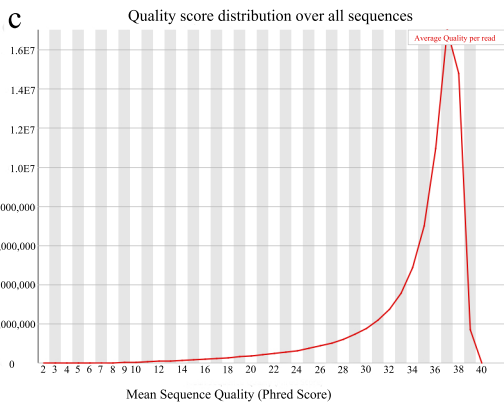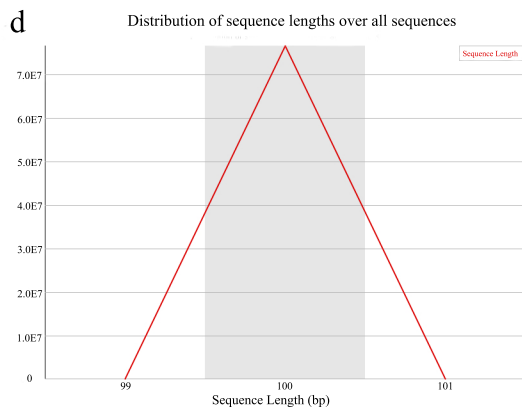

Supplement: Supplementary file 2 — Figure S2. Assessment of reads using FastQC after quality control. a) Quality of reads per base after adaptive window trimming using a quality average threshold of 20 and a minimum length threshold of 20. The central red line is the median value, the yellow box represents the interquartile range (25–75%), the upper and lower whiskers represent the 10 and 90% points, respectively, and the blue line represents the mean base quality. b) Sequence content across all bases. c) Distribution of the mean quality scores over all sequenced reads. d) Length distributions of all sequenced reads. (PDF 23882 kb) [file 12864_2019_5584_MOESM2_ESM.pdf]

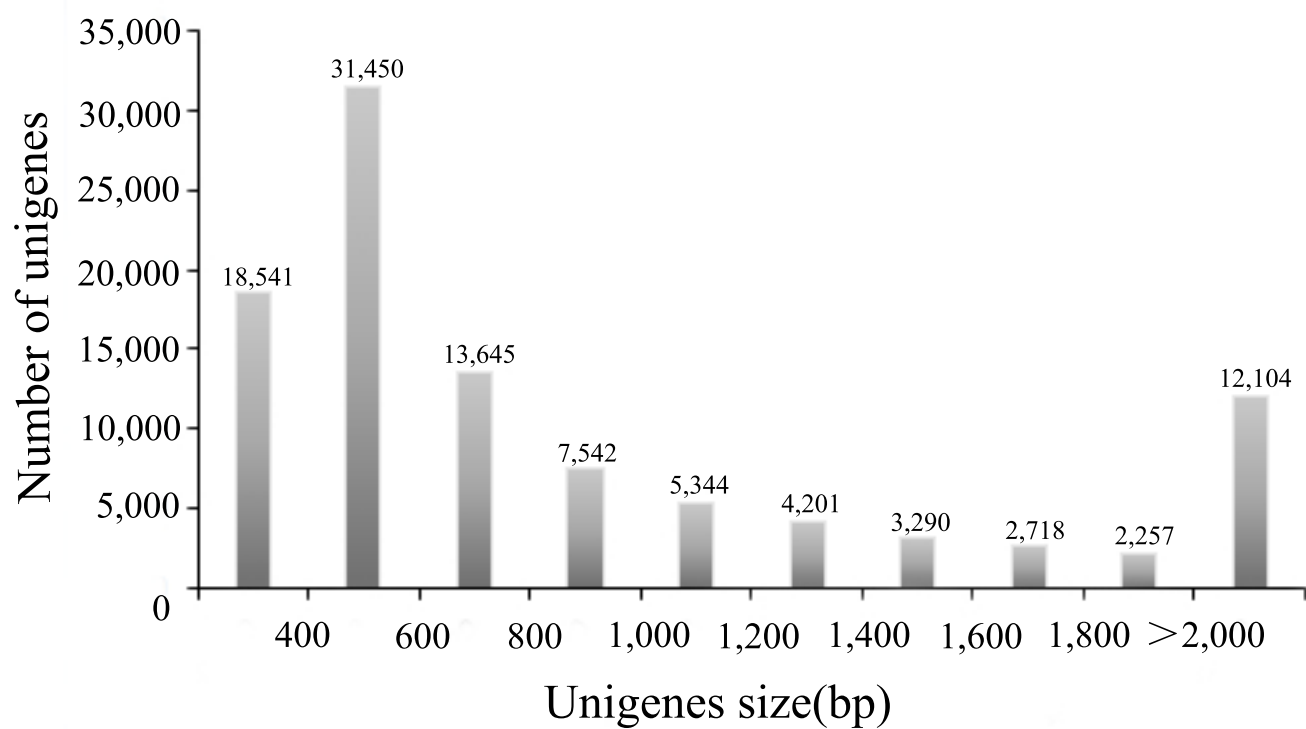

Supplement: Supplementary file 4 — Figure S3. Length distributions of all unigenes for C. gigantea. (PDF 596 kb) [file 12864_2019_5584_MOESM4_ESM.pdf]

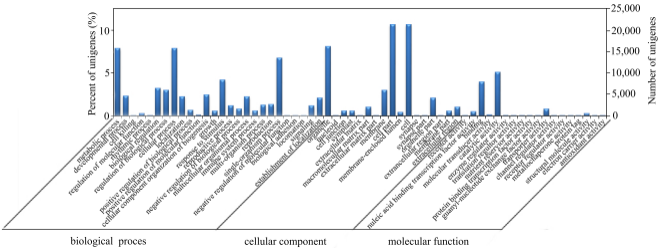

Supplement: Supplementary file 9 — Figure S4. GO annotation of C. gigantea unigenes. (PDF 2723 kb) [file 12864_2019_5584_MOESM9_ESM.pdf]

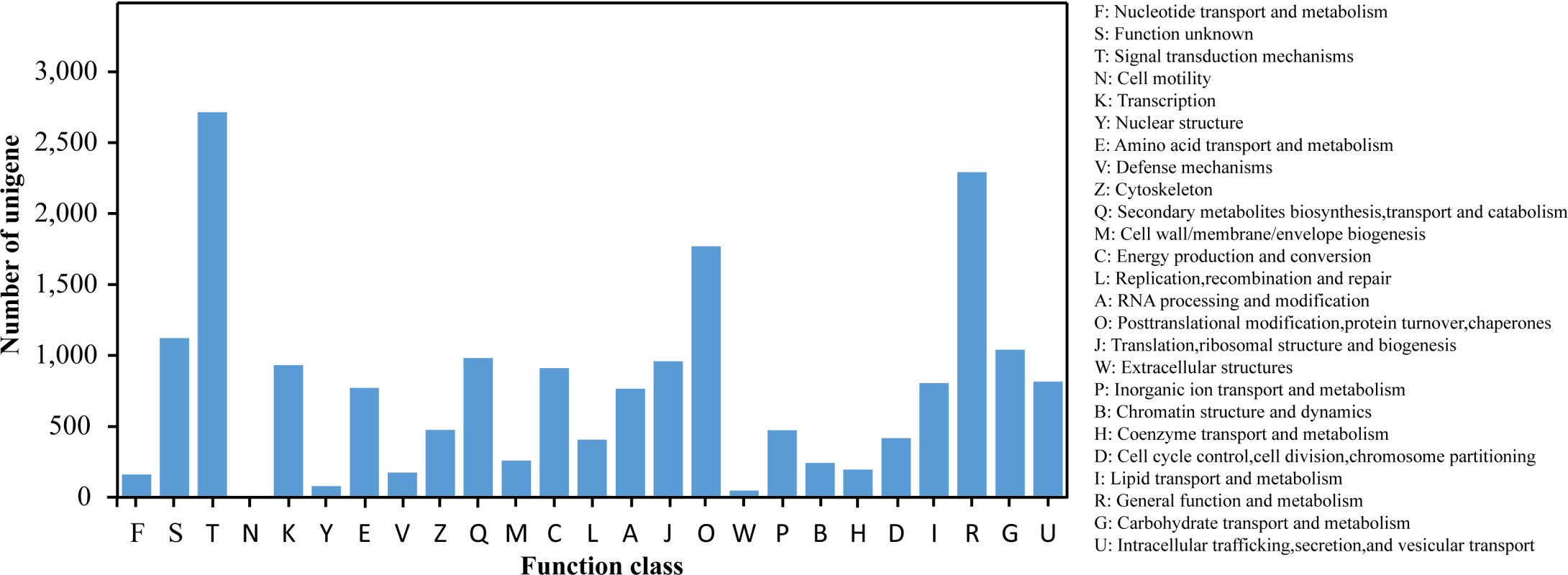

Supplement: Supplementary file 10 — Figure S5. KOG classification of C. gigantea unigenes. (PDF 1375 kb) [file 12864_2019_5584_MOESM10_ESM.pdf]

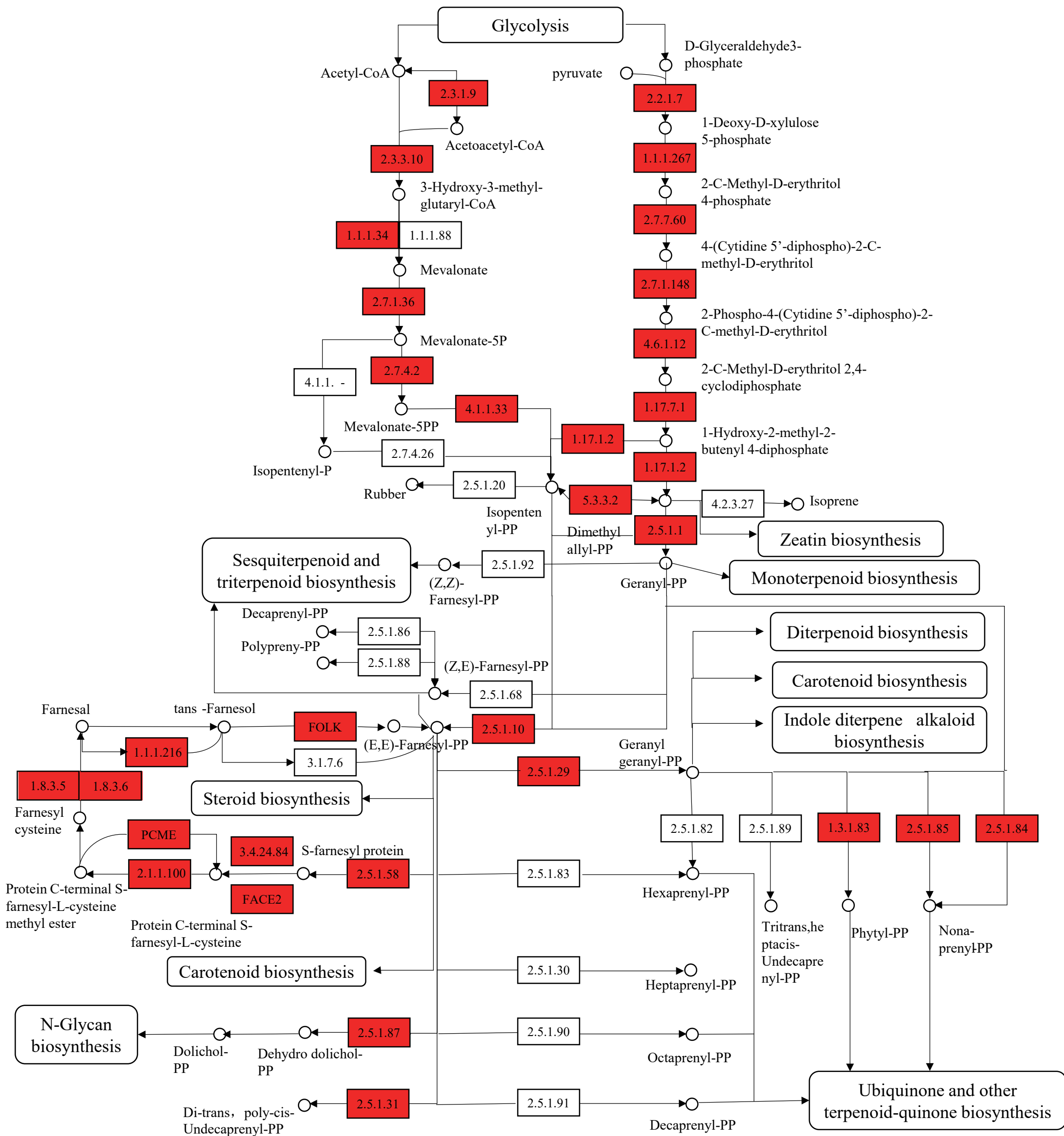

Supplement: Supplementary file 12 — Figure S6. Metabolic pathway of the Terpenoid bakcone biosynthesis for the unigenes identified in C. gigantea. Each box represents the substance involved in each section of the pathway. The red boxes represent substances assigned at least one unigene. (PDF 530 kb) [file 12864_2019_5584_MOESM12_ESM.pdf]

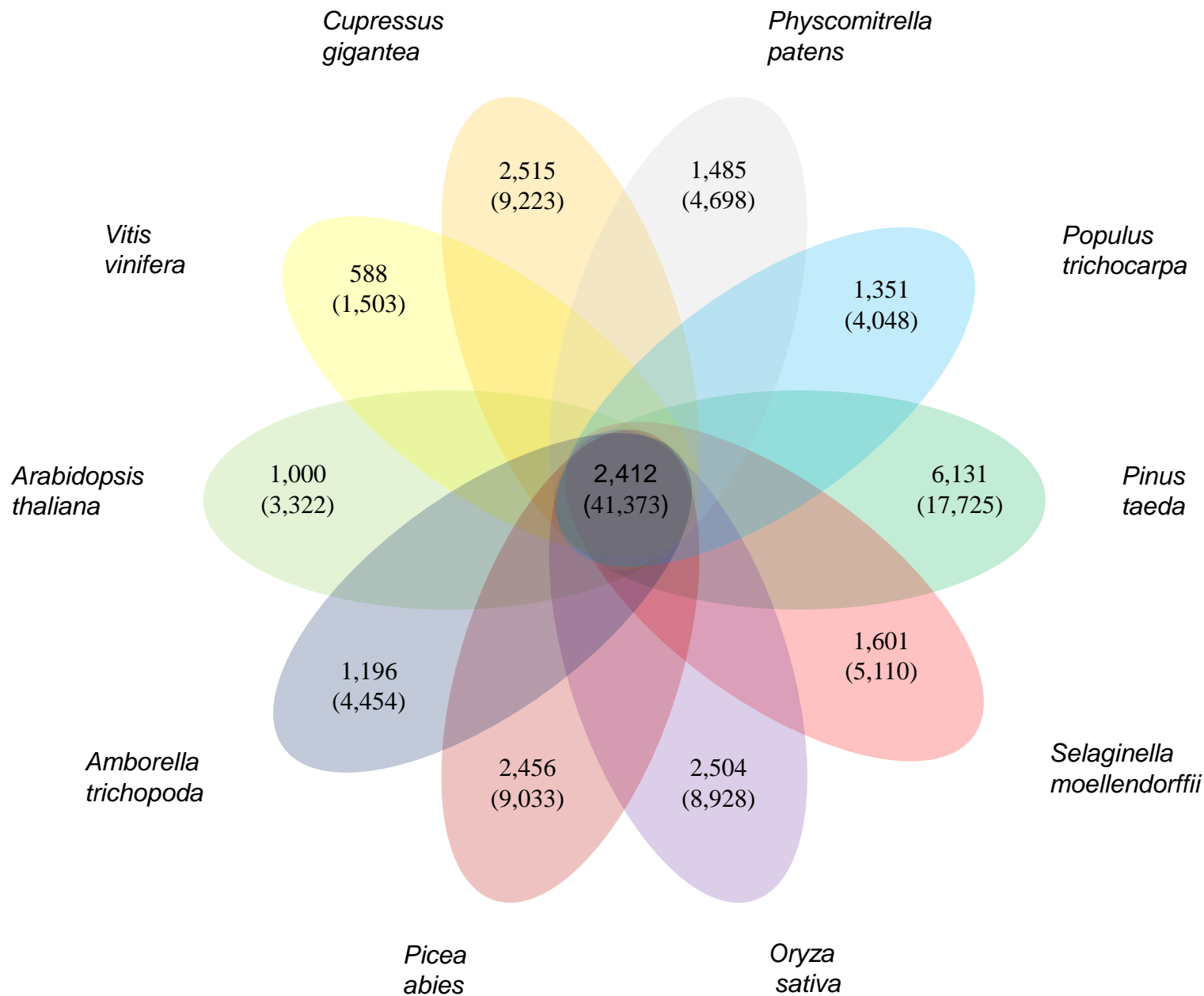

Supplement: Supplementary file 13 — Figure S7. Shared and unique gene families among C. gigantea and nine other plant species. (PDF 9 kb) [file 12864_2019_5584_MOESM13_ESM.pdf]

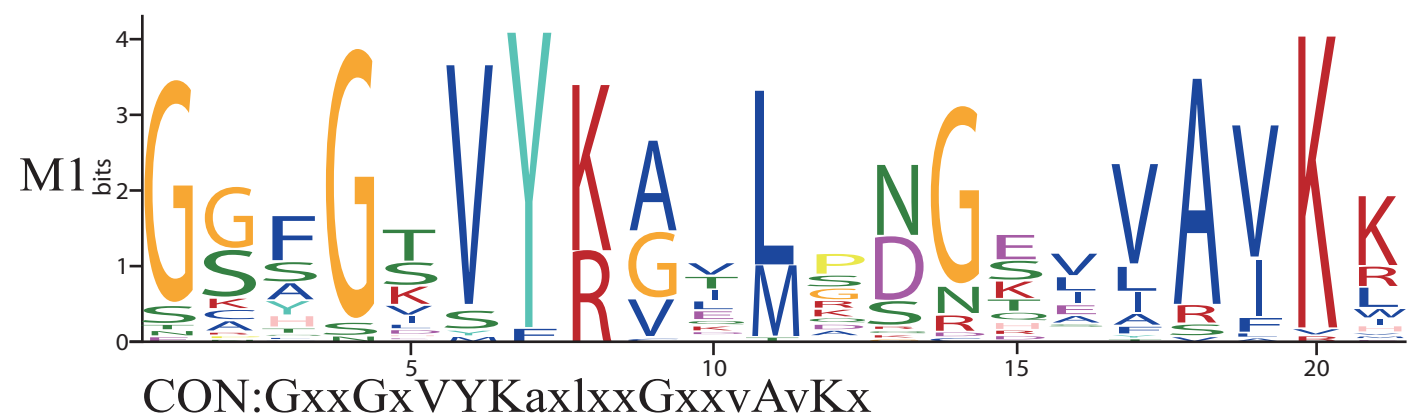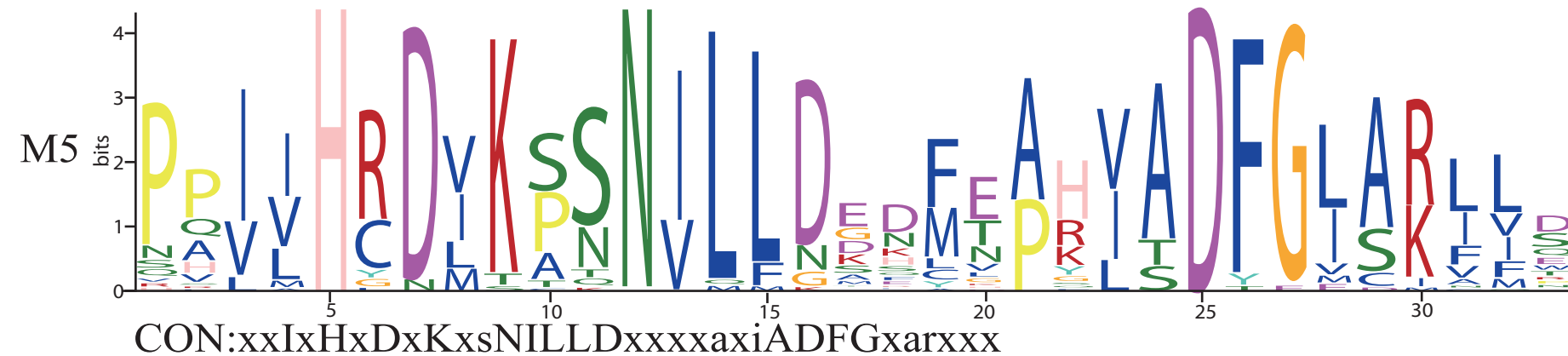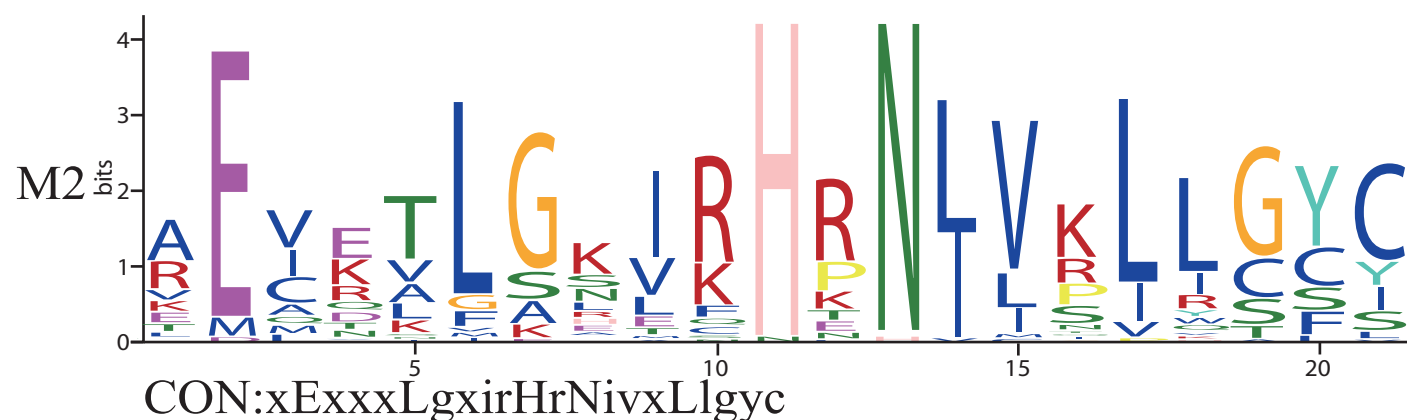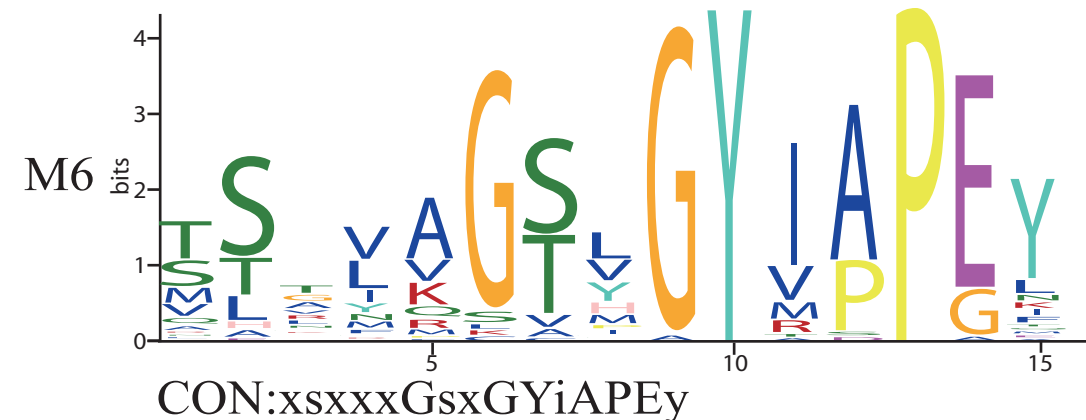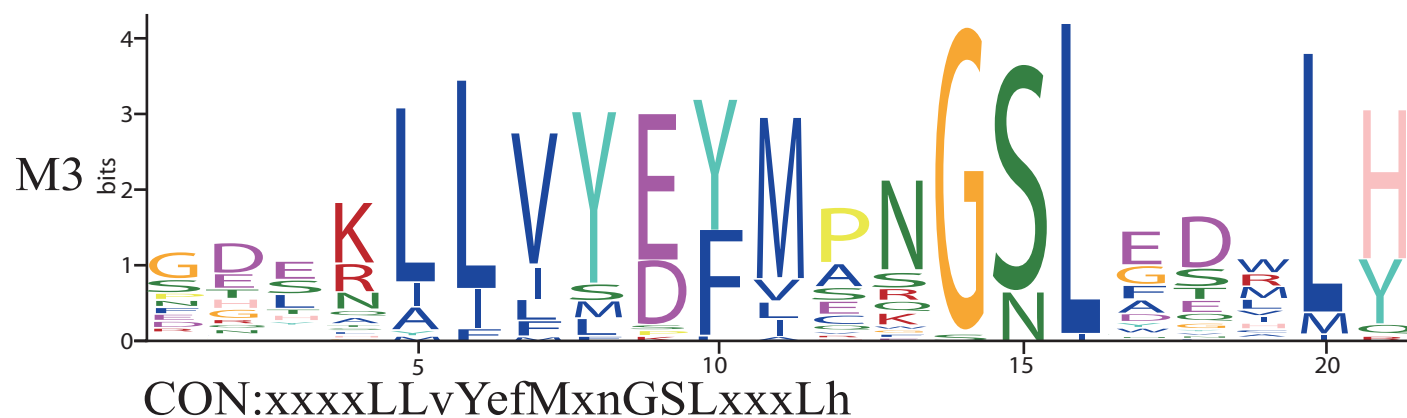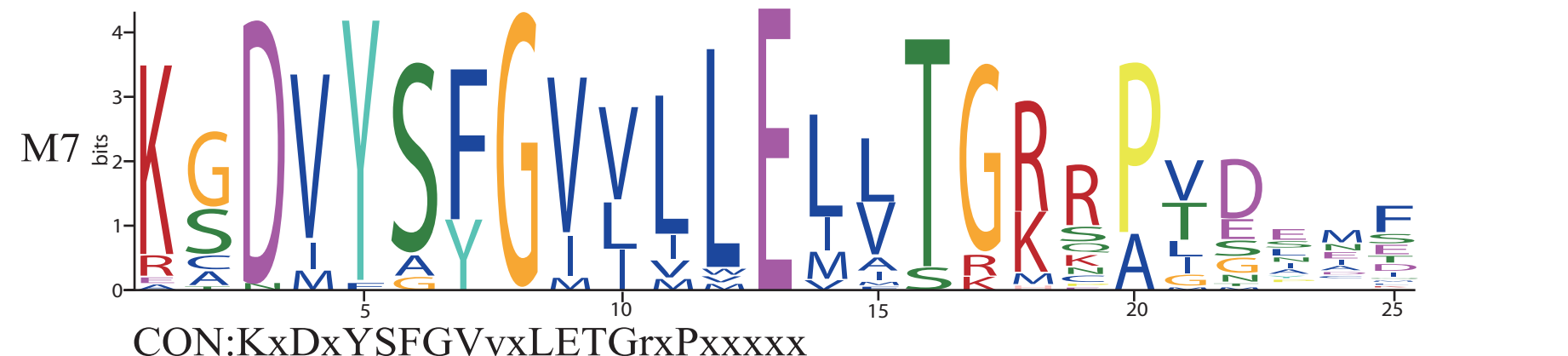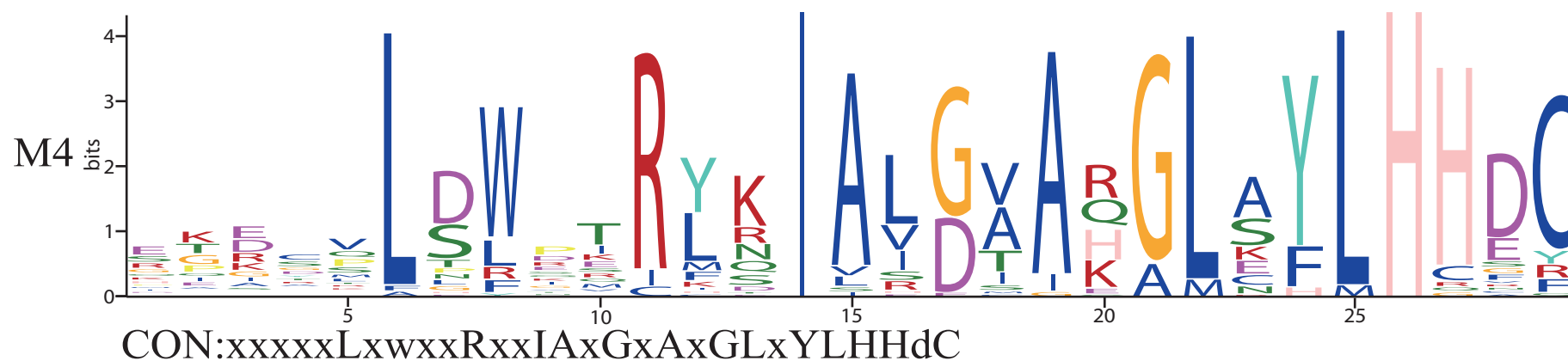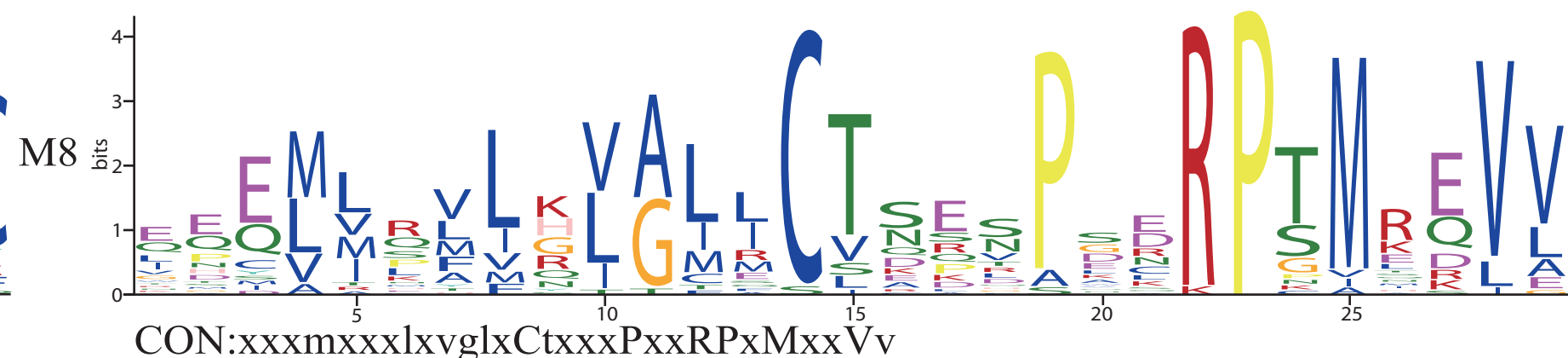

Supplement: Supplementary file 16 — Figure S9. Conserved motifs in LRR-RLK domain from C. gigantea transcriptome and their consensus sequences. Conserved motifs for the LRR-RLK domain from C. gigantea transcriptome and their consensus sequences. ‘CON’ indicates the consensus sequence. If the bits value of amino acid at this position is smaller than 1, it is represented with x; 2 > bits ≥1, with lowercase; 3 > bits ≥2, with capital letter; bits ≥3, with bold capital. (PDF 942 kb) [file 12864_2019_5584_MOESM16_ESM.pdf]

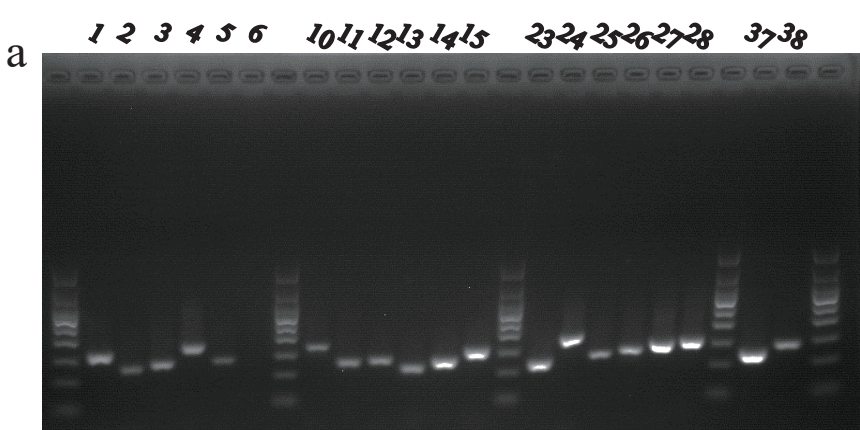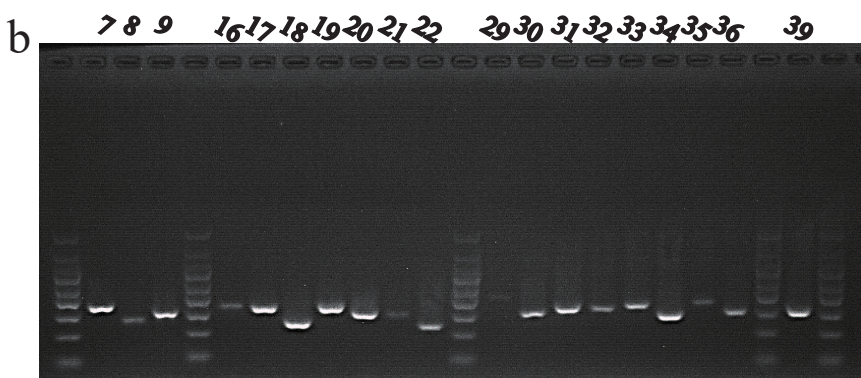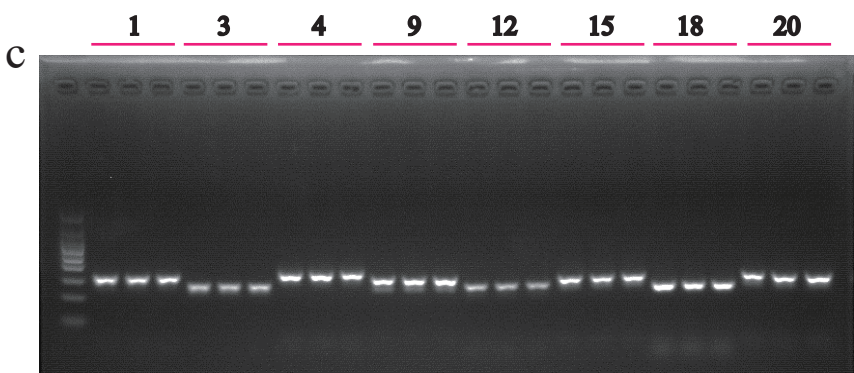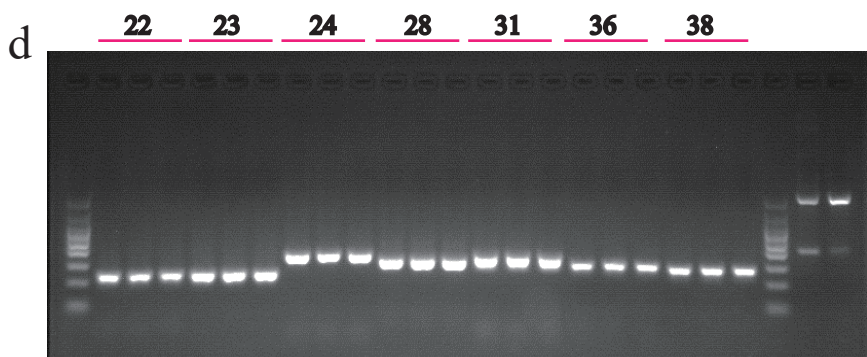

Supplement: Supplementary file 18 — Figure S11. Gel electrophoresis of 39 primers for real-time PCR with cDNA as template (a and b) and a subset (c and d) primers sets using cDNA as template for qRT-PCR. PCR was performed (d) with one primer pair failed to amplify target in RT-PCR using DNA as template (two columns on the most right side). The numbers on top of each plot (a, b, c and d) indicate the code of primer used for PCR amplification, refer to Supplementary S Table 9 for details of each primer pair. (PDF 870 kb) [file 12864_2019_5584_MOESM18_ESM.pdf]

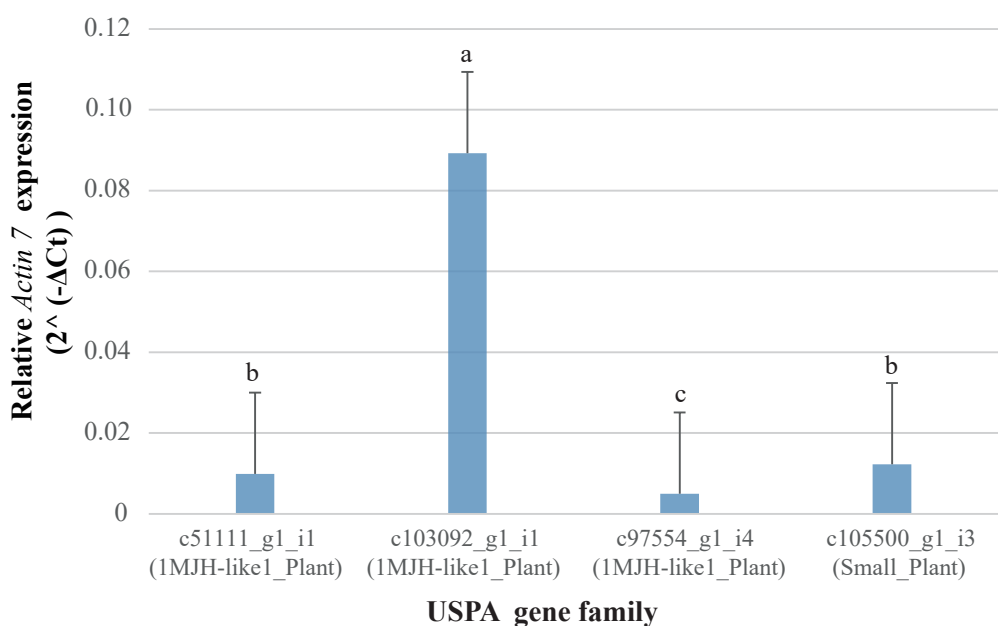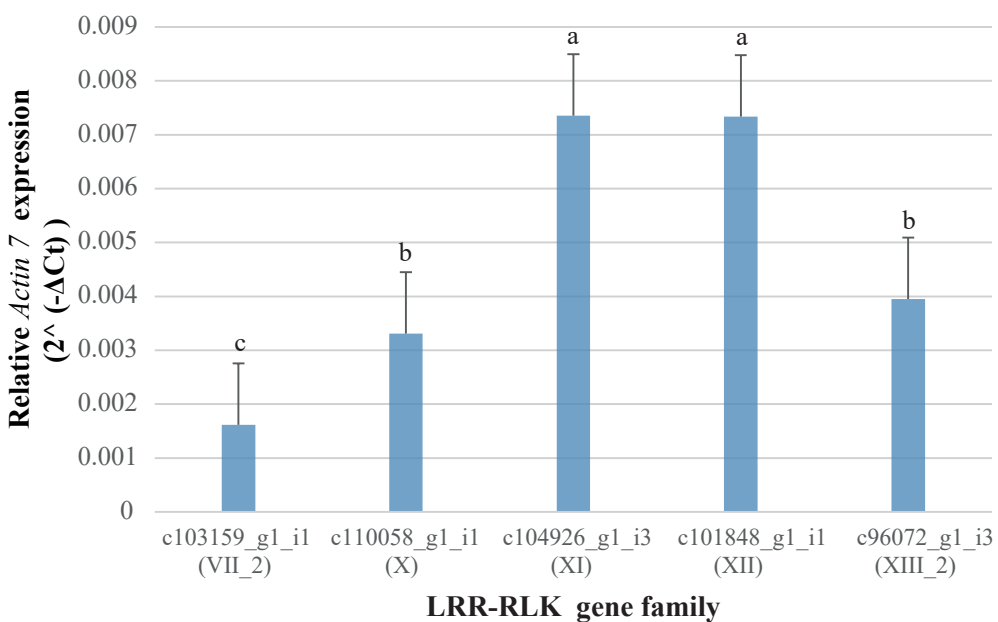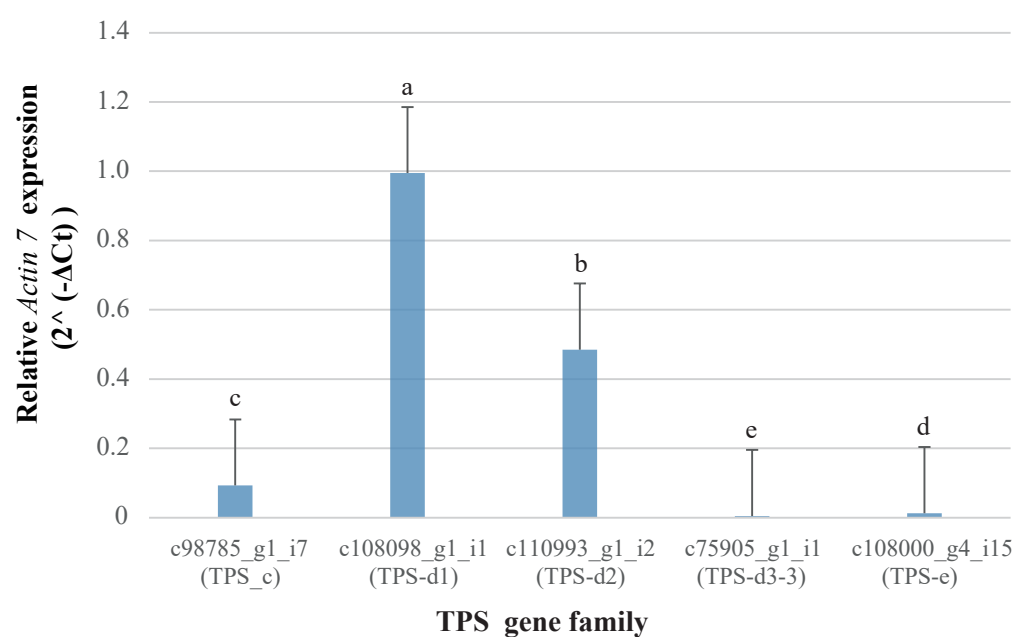

Supplement: Supplementary file 19 — Figure S12. Test of differential gene expresssion among selected genes from three gene families (USPA, LRR-RLK and TPS) based on quantitative real-time RT-PCR (qRT-PCR). Relative Actin gene expression and result of test on differential gene expression among different genes were shown. (PDF 386 kb) [file 12864_2019_5584_MOESM19_ESM.pdf]
