# Supplementary material for: In-depth transcriptome characterization uncovers distinct gene family expansions for Cupressus gigantea important to this long-lived species’ adaptability to environmental cues
Source: BMC Genomics. 2019 Mar 13;20:213. doi: 10.1186/s12864-019-5584-6 (PMC6417167; doi:10.1186/s12864-019-5584-6)
Supplement: Supplementary file 3 — Table S1. Summary of assembled contigs, scaffolds and unigenes properties for the C. gigantea unigenes. (DOCX 15 kb) [file 12864_2019_5584_MOESM3_ESM.docx]

**Supplementary Table 1. Summary of assembled contigs, scaffolds and unigenes properties for the *C. gigantea* unigenes.**

|  | contigs | scaffords | Unigenes |
| --- | --- | --- | --- |
| GC% | 38 | 38 | 38.03 |
| N25 (bp) | 2,284 | 2,877 | 2,906 |
| N50 (bp) | 1,136 | 1,482 | 1,508 |
| N75 (bp) | 533 | 670 | 680 |
| Counts | 135,542 | 102,553 | 101,092 |
| Max length (bp) | 18,728 | 23,390 | 25,331 |
| Average length (bp) | 764 | 1,018 | 1,029 |
| Total length (bp) | 103,584,408 | 104,432,740 | 104,109,640 |
