# Supplementary material for: In-depth transcriptome characterization uncovers distinct gene family expansions for Cupressus gigantea important to this long-lived species’ adaptability to environmental cues
Source: BMC Genomics. 2019 Mar 13;20:213. doi: 10.1186/s12864-019-5584-6 (PMC6417167; doi:10.1186/s12864-019-5584-6)
Supplement: Supplementary file 5 — Table S2. Summary of database matches (specific values) for C. gigantea unigenes. (DOCX 15 kb) [file 12864_2019_5584_MOESM5_ESM.docx]

**Supplementary Table 2. Summary of database matches (specific values) for *C. gigantea* unigenes.**

|  | Annotations | Percentage | Functional classification |
| --- | --- | --- | --- |
| All unigenes | 101,092 |  |  |
| Nr | 33,302 | 32.94% |  |
| Swiss-Port | 24,078 | 23.82% |  |
| KEGG | 6,848 | 6.77% | 292 pathways |
| KOG | 16,660 | 16.48% | 25 categories |
| GO | 28,087 | 27.78% | 3 main categories, 44 sub-categories |
| All annotated unigenes | 44,187 | 43.71% |  |
| Unigenes matching all five databses | 3,930 | 3.89% |  |
