# Supplementary material for: In-depth transcriptome characterization uncovers distinct gene family expansions for Cupressus gigantea important to this long-lived species’ adaptability to environmental cues
Source: BMC Genomics. 2019 Mar 13;20:213. doi: 10.1186/s12864-019-5584-6 (PMC6417167; doi:10.1186/s12864-019-5584-6)
Supplement: Supplementary file 15 — Figure S8. Multiple sequence alignment (MSA) of the USPA-like domains of the putative USPA protein. The annotation was done based on conserved features of 1MJH secondary structure (five β strands and four α helices). (PDF 6074 kb) [file 12864_2019_5584_MOESM15_ESM.pdf]

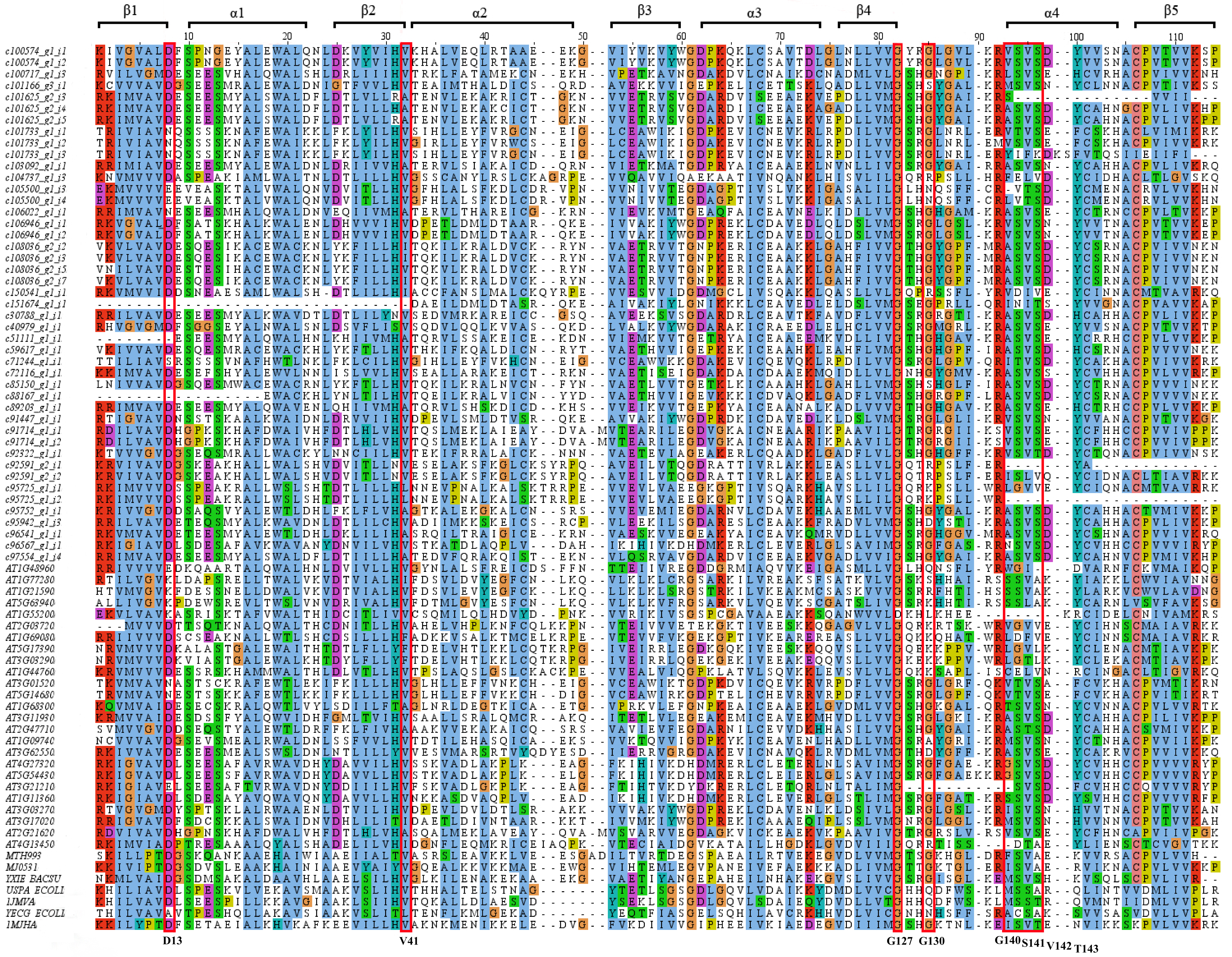
β1
α1
β2
α2
β3
α3
β4
α4
β5
D13
V41
G127
G130
G140
S141
V142
T143
