# Supplementary material for: In-depth transcriptome characterization uncovers distinct gene family expansions for Cupressus gigantea important to this long-lived species’ adaptability to environmental cues
Source: BMC Genomics. 2019 Mar 13;20:213. doi: 10.1186/s12864-019-5584-6 (PMC6417167; doi:10.1186/s12864-019-5584-6)
Supplement: Supplementary file 17 — Figure S10. Alignment of the conserved RxR and DDxxD motifs and motifs variation among 121 TPS sequences from C. gigantea transcriptome, with their corresponding consensus sequences. Multiple sequence alignment of the TPS domain sequences from C. gigantea transcriptome. Conserved motifs for the TPS domain from C. gigantea transcriptome and their consensus sequences. ‘CON’ indicates consensus sequence. If the bits value of amino acid at this position was smaller than 1, it was represented with x; 2 > bits ≥1, with lowercase; 3 > bits ≥2, with capital letter; bits ≥3, with bold capital. (PDF 7804 kb) [file 12864_2019_5584_MOESM17_ESM.pdf]

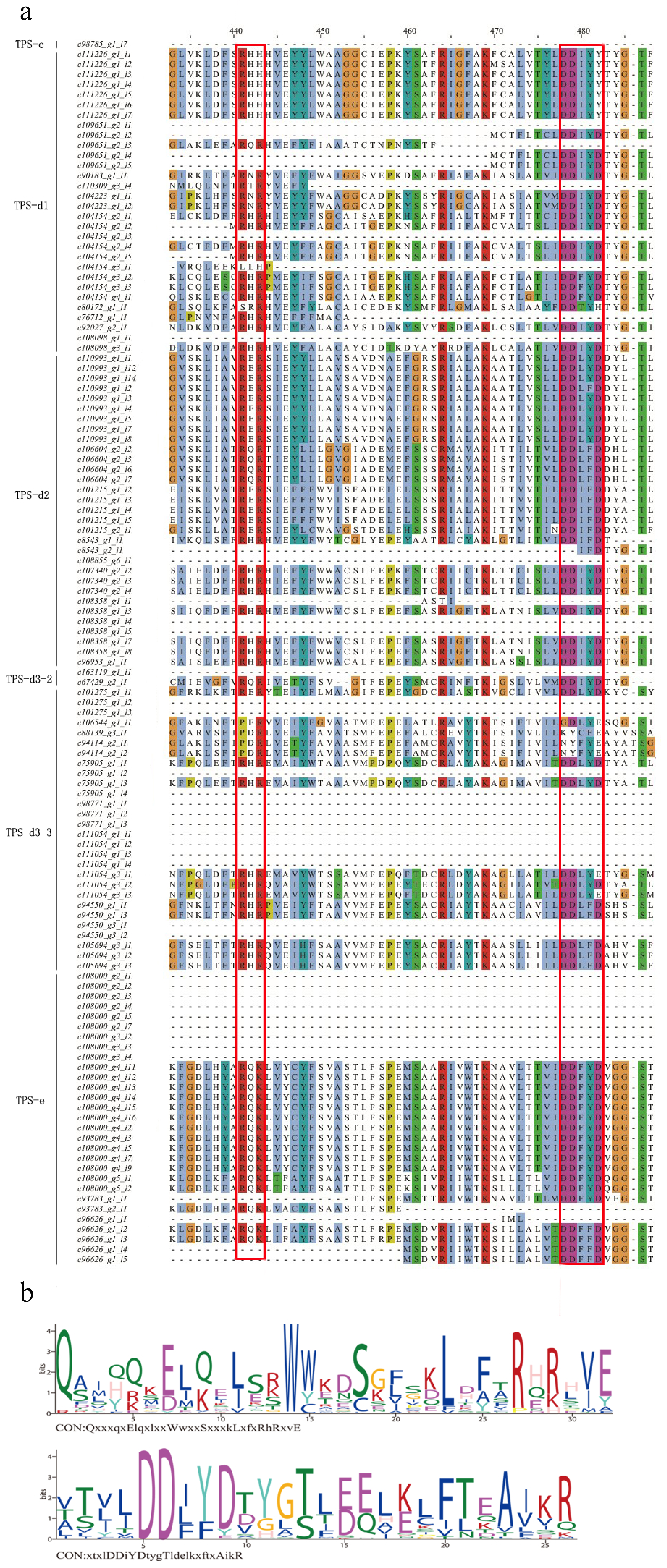
a
440
450
460
470
480
TPS-c
TPS-d1
TPS-d2
TPS-d3-2
TPS-d3-3
TPS-e
b
CON:QxxxqxElqlxxWwxxSxxxkLxfxRhRxvE
CON:xtxlDDiYDtygTldelkxftxAikR
