# Supplementary material for: In-depth transcriptome characterization uncovers distinct gene family expansions for Cupressus gigantea important to this long-lived species’ adaptability to environmental cues
Source: BMC Genomics. 2019 Mar 13;20:213. doi: 10.1186/s12864-019-5584-6 (PMC6417167; doi:10.1186/s12864-019-5584-6)
Supplement: Supplementary file 21 — Table S9. Sequences of primers for real-time PCR. (DOCX 23 kb) [file 12864_2019_5584_MOESM21_ESM.docx]

**Supplementary Table 9. Primers used for PCR and qRT-PCR**.

| Number | Gene ID | Primer(5' → 3') | Length (nt) | Product size (bp) | Subfamilies | Families |
| --- | --- | --- | --- | --- | --- | --- |
| 1 | c51111_g1_i1* | F: TGACAGGAGAAACACGGTATG | 21 | 161 | 1MJH-like_Plant | USPA |
|  |  | R: GACAACTAAGACTGGGCACTT | 21 |  |  |  |
| 2 | c96541_g1_i1 | F: TTGGCATTGTGCGAACAGTAA | 21 | 123 | 1MJH-like_Plant |  |
|  |  | R: TGCGATATGTGAGGCGGTTAA | 21 |  |  |  |
| 3 | c103092_g1_i1* | F: CTGTAATAGGAAGCGTGAGTA | 21 | 136 | 1MJH-like_Plant |  |
|  |  | R: AGATCACAGCCAAGATAAGAA | 21 |  |  |  |
| 4 | c97554_g1_i4* | F: TGCACGCTCAACCGCTTCCTA | 21 | 177 | 1MJH-like_Plant |  |
|  |  | R: CAGCCACTCTGCTCTGTAAACAC | 23 |  |  |  |
| 5 | c101625_g2_i4 | F: GAGCGTGGAGGAGAATAAGGG | 21 | 133 | 1MJH-like_Plant |  |
|  |  | R: GTGGACGGCAGTAAACAGAGG | 12 |  |  |  |
| 6 | c106946_g1_i2 | F: ACGATTTCCCACTTCCTTCAA | 21 | 170 | 1MJH-like_Plant |  |
|  |  | R:AACGCCTACCTTTCTATCTCCTG | 23 |  |  |  |
| 7 | c92591_g2_i1 | F: AAACTGAAGGCACAATACACGC | 22 | 173 | Small_Plant |  |
|  |  | R: CATCGACTGCCACAATTACCC | 21 |  |  |  |
| 8 | c95725_g1_i2 | F: GAGTAGTATTGGGATGGGTAG | 21 | 129 | Small_Plant |  |
|  |  | R: AGTAGGGATTCACAAGAACTG | 21 |  |  |  |
| 9 | c105500_g1_i3* | F: AGTAATGGCAGTGGAGAAGAT | 21 | 154 | Small_Plant |  |
|  |  | R: CTTGTGACCTGGAGTTGTTTC | 21 |  |  |  |
| 10 | c110005_g1_i5 | F: TCCCACGGGTTTGGAGAAGTT | 21 | 172 | III | LRR-RLK |
|  |  | R: GCTGGCTATGATTTGCATTTT | 21 |  |  |  |
| 11 | c66588_g1_i1 | F: TGTTACCAGTCCTAATGCTCCTT | 23 | 128 | VII_1 |  |
|  |  | R: CGGTTAATCTCCCAATCTCCT | 21 |  |  |  |
| 12 | c103159_g1_i1* | F: GCTGTGATAGCGACAGTGTATG | 22 | 133 | VII_2 |  |
|  |  | R: TCCACGACCTAAGAAACGAAT | 21 |  |  |  |
| 13 | c102164_g1_i1 | F: ATTGATGGAAGGGTGAAAGGG | 21 | 120 | X |  |
|  |  | R: GCTGAAACGGTTATGGTGGAA | 21 |  |  |  |
| 14 | c108144_g1_i1 | F:GCAAACATCAGAACCCTCAAC | 21 | 136 | X |  |
|  |  | R: TGCAAACGGTGGTAGGAATAG | 21 |  |  |  |
| 15 | c110058_g1_i1* | F: CGGGTTTGGAGATTGCGTGAG | 21 | 155 | X |  |
|  |  | R: CCCATAATGAAGCGGGTAGTT | 21 |  |  |  |
| 16 | c94622_g1_i1 | F: TCTCACTCAGTTGGTCGTTGT | 21 | 165 | XI |  |
|  |  | R: AGATACTCCAGCCACTTCAGC | 21 |  |  |  |
| 17 | c100696_g1_i1 | F: CTTTCCAAGCCTTACTACTCTG | 22 | 168 | XI |  |
|  |  | R: TAAATCCATTCAAGCCTCCTG | 21 |  |  |  |
| 18 | c104926_g1_i3* | F: CTCAGGAGGCATACCCAACCA | 21 | 131 | XI |  |
|  |  | R: GTGCGACAGTCAGACACGACA | 21 |  |  |  |
| 19 | c96588_g1_i1 | F: CGGGCTTACAAATGCTCTGGC | 21 | 177 | XII |  |
|  |  | R: GGTTATGACGAAGGCGTAGCG | 21 |  |  |  |
| 20 | c101848_g1_i1* | F: ATCGTTCATTGTATCAGCAGGAG | 23 | 159 | XII |  |
|  |  | R: AAAGCACCGCACTTTACACCC | 21 |  |  |  |
| 21 | c111059_g6_i1 | F: TCCGAACGAAATAGGGAGAAT | 21 | 147 | XII |  |
|  |  | R: GGAATAGTGCCAACCAGTAGA | 21 |  |  |  |
| 22 | c96072_g1_i3* | F: GGATTGACGCCAACTCTAACG | 21 | 122 | XIII_2 |  |
|  |  | R: TGCTGGTGATATTTCTCCTCC | 21 |  |  |  |
| 23 | c98785_g1_i7* | F: ACATCAAGTCTACGCCGAAAG | 21 | 121 | TPS_C | TPS |
|  |  | R: ATCCCAATCTACCACATCACG | 21 |  |  |  |
| 24 | c108098_g1_i1* | F: CACCACCACTCTTGAAACACC | 21 | 175 | TPS-d1 |  |
|  |  | R: TGTGGGCATCTGATTGAACAT | 21 |  |  |  |
| 25 | c109651_g2_i2 | F: GGCCCTCAGACTAAGAAATGA | 21 | 129 | TPS-d1 |  |
|  |  | R: AGATACTCTAATGCTTCCTCC | 21 |  |  |  |
| 26 | c111226_g1_i2 | F: CGCCACAGCTTTGGGTTTTCG | 21 | 139 | TPS-d1 |  |
|  |  | R: AATGCTTTTCATCGCCTCCTC | 21 |  |  |  |
| 27 | c101215_g2_i1 | F: CGACAACGGGCAGTTCATCCT | 21 | 153 | TPS-d2 |  |
|  |  | R: GCTTCAGCCTCCTCCATCACG | 21 |  |  |  |
| 28 | c110993_g1_i2* | F: CCCATCCTCCTAGCAGTTCCT | 21 | 162 | TPS-d2 |  |
|  |  | R: AATTGCCGACGCTGTTTGTCC | 21 |  |  |  |
| 29 | c110993_g1_i3 | F: CATTCCACCTGATCCTCCTGG | 21 | 170 | TPS-d2 |  |
|  |  | R: AATTGCTGACGCTGTTTGTCC | 21 |  |  |  |
| 30 | c163119_g1_i1 | F: GTCTTGTTCCCTGTGCTTTGC | 21 | 155 | TPS-d3-2 |  |
|  |  | R: CTCCTCAGCCTCAGAAGTTGC | 21 |  |  |  |
| 31 | c75905_g1_i1* | F: AGAGGCATTGGTTGCGGAGAT | 21 | 174 | TPS-d3-3 |  |
|  |  | R: CCGTCGGGTAACTGGTTGTGA | 21 |  |  |  |
| 32 | c105694_g3_i1 | F: CCTGCGTCTTCCTACATACTGG | 22 | 160 | TPS-d3-3 |  |
|  |  | R: AAATAGCGGTCAATCCCTAAA | 21 |  |  |  |
| 33 | c111054_g1_i1 | F: AACCCTAATCACGGGAAACCA | 21 | 171 | TPS-d3-3 |  |
|  |  | R: AACCCAGGCAGTATCGTAAGC | 21 |  |  |  |
| 34 | c96626_g1_i4 | F: CACCCAGAAGCATCTGTTGAG | 21 | 151 | TPS-e |  |
|  |  | R: GCTGGATGAGTTTGAGCGTAG | 21 |  |  |  |
| 35 | c108000_g2_i1 | F: TTCACTCTTCCATTCTCCCTC | 21 | 172 | TPS-e |  |
|  |  | R: TAGCCTTTCCAACTTATCCAC | 21 |  |  |  |
| 36 | c108000_g4_i15* | F: TGAGTATGCACTGGCTGTACC | 21 | 150 | TPS-e |  |
|  |  | R: GCTAATGTCGAAAAGATGTCA | 21 |  |  | |
| 37 | a18s | F: CGAGACCTCAGCCTGCTAACT | 21 | 128 | Internal reference gene | |
|  |  | R: AGAACATCTAAGGGCATCACA | 21 |  |  | |
| 38 | bActin 7* | F: TCACCAGAATCCAGCACAATA | 21 | 142 |  | |
|  |  | R: TGAACCCTAAGGCAAACAGAG | 21 |  |  | |
| 39 | bUbiquitin 10 | F: ATCCACGCTCCACCTTGTGCT | 21 | 158 |  | |
|  |  | R: GCTGGTCTGGCGGAATACCCT | 21 |  |  | |

F and R indicate forward and reverse primers, respectively.

* indicate primers used for determination of gene expression with qRT-PCR.

^a^ indicate primers for amplification of internal reference genes designed from *Cupressus gigantea*.

^b^ indicate primers for amplification of internal reference genes designed with homologous gene from *Arabidopsis thalian*.
